# Comparing outcomes between coronary artery bypass grafting and percutaneous coronary intervention in octogenarians with left main or multivessel disease

**DOI:** 10.1038/s41598-023-49069-2

**Published:** 2023-12-15

**Authors:** Hristo Kirov, Tulio Caldonazo, Leoni Lu Riedel, Panagiotis Tasoudis, Alexandros Moschovas, Mahmoud Diab, Gloria Färber, Torsten Doenst

**Affiliations:** 1 Department of Cardiothoracic Surgery, Friedrich-Schiller-University, Jena, Germany; 2 Division of Cardiothoracic Surgery, University of North Carolina, Chapel Hill, USA; 3 Department of Cardiothoracic Surgery, Friedrich-Schiller-University Jena, Am Klinikum 1, 07747 Jena, Germany

**Keywords:** Cardiovascular biology, Interventional cardiology, Epidemiology

## Abstract

Mechanisms of coronary artery bypass grafting (CABG) and percutaneous coronary intervention (PCI) differ as CABG provides surgical collateralization and may prolong life by preventing future myocardial infarctions (MI). However, CABG benefits are unclear in octogenarians, where surgical risk is often perceived as higher and PCI is chosen more liberally. We performed a meta-analysis of studies comparing outcomes in octogenarians with left main or multivessel disease who underwent CABG or PCI. Primary outcome was late mortality (> 5 years). Secondary outcomes were perioperative mortality, MI, re-revascularization (R-R), acute renal failure (ARF), and stroke. Fourteen studies with 17,942 patients were included. CABG was associated with lower late mortality (hazard ratio, HR: 1.23, 95% confidence interval: CI 1.05–1.44, p < 0.01). In the pooled Kaplan–Meier analysis CABG showed significantly lower risk of death in the follow-up compared to PCI (HR: 1.08, 95%CI 1.02–1.41, p = 0.005). Landmark analyses confirmed the survival advantage of CABG over PCI after 21.5 months of follow-up (HR: 1.31, 1.19–1.44, p < 0.0001), but suggested advantage of PCI over CABG in the first 30-days (HR: 0.72, 0.64–0.82, p < 0.0001) and comparable survival from 1 to 21.5 months (HR: 0.98, 0.92–1.05, p = 0.652). We found lower risk for MI and R-R after CABG but higher perioperative mortality and no differences in ARF and stroke. CABG appears superior to PCI over time in octogenarians with complex CAD. This survival advantage is associated with fewer events of MI and R-R; however, it comes with an increased risk in perioperative mortality.

## Introduction

Recent data suggest that mechanisms of coronary artery bypass grafting (CABG) surgery and percutaneous coronary intervention (PCI) differ^1^. Guideline-conform PCI is focused on treating flow-limiting lesions, but the majority of myocardial infarctions occur at non-flow-limiting stenoses^1^. Thus, PCI cannot be expected to significantly limit new myocardial infarctions (MI). In contrast, CABG may do so by bypassing most coronary lesions providing downstream “collateralization” to the grafted vessel and possibly to other coronary arteries, which may prevent myocardial infarctions caused by ruptured plaque thrombosis or sudden progression of plaques that were not flow limiting at the time of surgery^1,2^. This potential mechanism was confirmed by a recent meta-analysis of all randomized studies comparing PCI and CABG, which showed that the observed survival advantage of CABG over PCI in randomized trials was associated with a significant reduction of spontaneous MIs in the surgical arm^3^.

As one ages, the likelihood of experiencing an acute MI progressively increases. In the United States, individuals aged 65 and older account for over 60% of all cases of acute MI, with approximately one third of cases occurring in those over 75 years old^4^. The mortality rates following an acute MI also increase significantly with age. This means that about 60% of all MI-related deaths in the United States occur among the 6% of the population aged 75 years or older^4^.

Due to the increased risk of MIs in octogenarians it might be well conceivable that surgical collateralization through CABG might be the superior invasive treatment. However, in those patients surgical risk is often perceived as prohibitively high and PCI is chosen more liberally. So far, there is no clear recommendation addressing the invasive treatment options for coronary artery disease in octogenarians. Therefore, in this analysis we set out to systematically review the literature on the impact of the invasive treatment modality on clinical outcome in octogenarians with coronary heart disease.

## Methods

Ethical approval of this analysis was not required as no human or animal subjects were involved. This review was registered with the National Institute for Health Research International Registry of Systematic Reviews (PROSPERO, CRD42022345249).

### Search strategy

We performed a comprehensive literature search to identify contemporary studies reporting short-, mid- and long-term outcomes between CABG and PCI in octogenarians with left main or multivessel coronary disease. Searches were run on June, 2022 in the following databases: Ovid MEDLINE (2008 to present); Web of Science (2012 to present); and The Cochrane Library (1993 to present). The search strategy for Ovid MEDLINE is available in Supplementary Table 1.

### Study selection

The study selection followed the Preferred Reporting Items for Systematic Reviews and Meta-Analyses (PRISMA) strategy. After de-duplication, records were screened by two independent reviewers (TC and LR). Any discrepancies and disagreements were resolved by a third author (HK). Titles and abstracts were reviewed against pre-defined inclusion and exclusion criteria.

### Eligibility criteria

Studies were considered for inclusion if they were written in English and reported direct comparison between CABG and PCI in octogenarians with left main or multivessel coronary disease. Animal studies, abstracts, case reports, commentaries, editorials, expert opinions, conference presentations, and studies not reporting the outcomes of interest were excluded. The full text was pulled for the selected studies for a second round of eligibility screening. References for articles selected were also reviewed for relevant studies not captured by the original search.

### Risk of bias assessement and data extraction

The Risk of Bias in Non-Randomized Studies of Interventions tool (ROBINS-I) was systematically used to assess included studies for risk of bias^5^. The studies and their characteristics were classified into low, moderate and serious risk of bias. Two independent reviewers (TC and LR) assessed risk for bias. When there was a disagreement, a third reviewer (HK) checked the data and made the final decision (Supplementary Fig. 1).

Two reviewers (TC and LR) independently performed data extraction. Accuracy was verified by a third author (HK). The extracted variables included study characteristics (publication year, country, sample size, study design, mean follow-up, presence or absence from population adjustment and outcome definitions) as well as patient demographics (age, sex, mean left ventricular ejection fraction—LVEF, hypertension, diabetes, smoking status, prior cerebrovascular accident—CVA, prior myocardial infarction MI, prior PCI, renal failure and chronic obstructive pulmonary disease—COPD).

### Outcomes

Primary outcome was long-term all-cause mortality defined by studies with follow-up > 5 years. Secondary outcomes were perioperative all-cause mortality (30-day/in-hospital), acute renal failure, myocardial infarction, re-revascularization and stroke.

### Statistical analysis

We conducted meta-analyses to compare the outcomes of CABG versus PCI. Relative risks (RR) and 95% confidence intervals (CI) were calculated for each outcome. A RR greater than 1 indicated that the outcome was more frequently present in the CABG arm. Inherent clinical heterogeneity between the studies was balanced via the implementation of a random effects models. Results were displayed in forest plots. Between-study statistical heterogeneity was assessed with the Cochran Q statistic and by estimating I2. High heterogeneity was confirmed with a significance level of p < 0.10 and I2 of at least 50% or more. Publication bias was assessed via funnel plots and Eggers’ test for the primary outcome and p < 0.10 was considered statistically significant. Leave-one-out sensitivity analyses were also performed for the primary outcome. All analyses were performed using STATA IC17.0 (StataCorp LLC, College Station, Texas).

### Reconstruction of individual patient survival data

We used the methods described by Wei et al. to reconstruct IPD from the Kaplan–Meier curves of all eligible studies for the long-term outcomes^6,7^. Raster and Vector images of the Kaplan–Meier survival curves were pre-processed and digitized, so that the values reflecting to specific timepoints with their corresponding survival/mortality information could be extracted. Where additional information (e.g., number-at-risk tables or total number of events) were available, they were used to further calibrate the accuracy of the time-to-events. Departures from monotonicity were detected using isotonic regression and corrected with a pool-adjacent-violators algorithm^6,7^. To confirm the quality of the timing of failure events captured, we thoroughly checked the consistency with the reported survival or morality data provided in the original publications.

### meta-analysis of reconstructed data—one-stage survival meta-analysis

The Kaplan–Meier method was used to calculate the overall survival. The Cox proportional hazards regression model was used to assess between-group differences. For these Cox models, the proportional hazards assumption was verified by plotting scaled Schoenfeld residuals, log–log survival plots, and predicted versus observed survival functions. We plotted survival curves using the Kaplan–Meier product limit method and calculated the Hazard Ratios (HRs) and 95% CIs of each group. A HR greater than 1 indicated that the outcome was more frequently present in the PCI arm.

### Presentation

This work has been selected to be presented at the 103rd AATS Annual Meeting.

## Results

### Study characteristics

**Figure 1:**
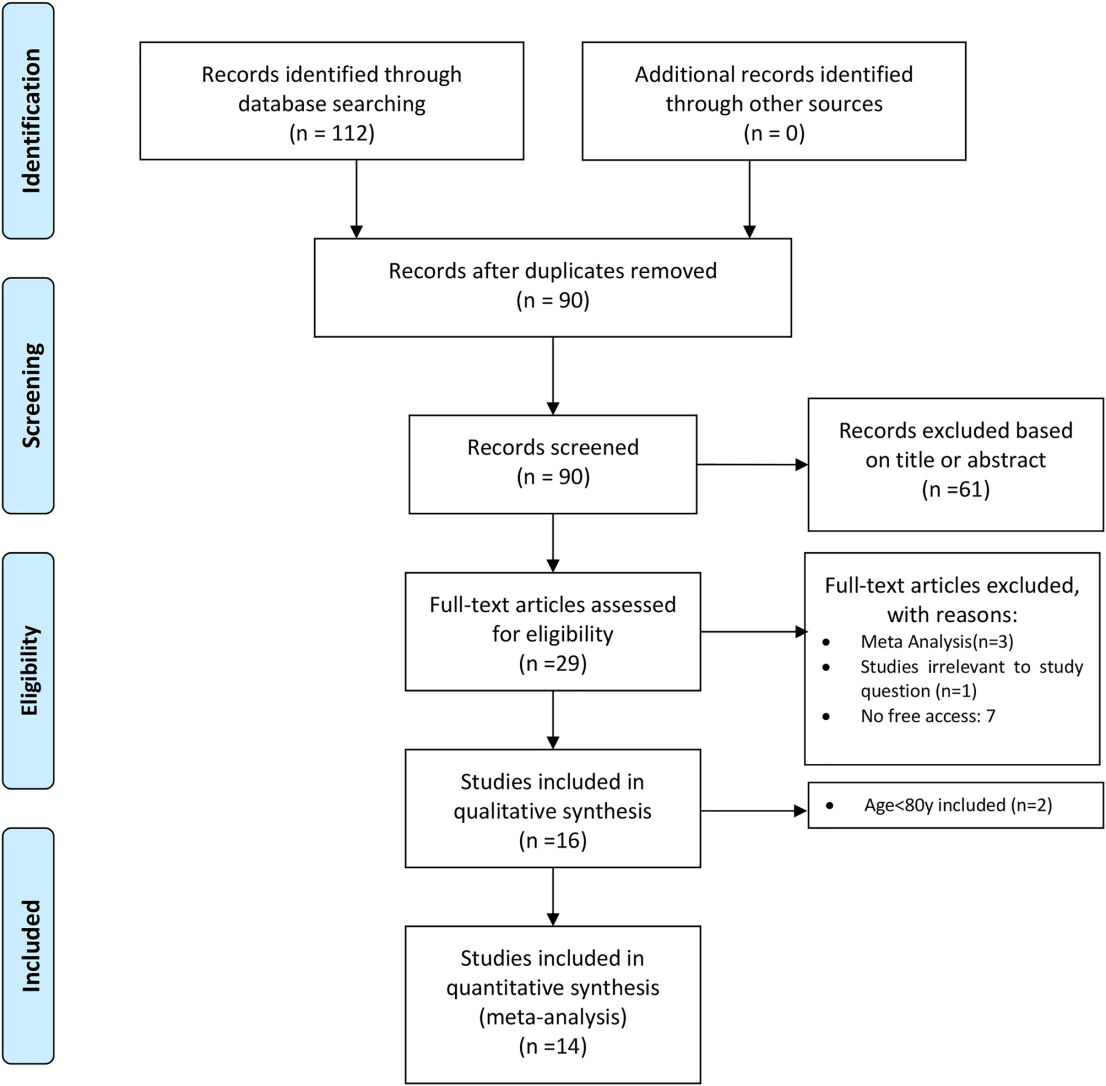
Preferred Reporting Items for Systematic Reviews and Meta-Analyses (PRISMA) flow diagram.

A total of 112 studies were retrieved from the systematic search, of which 14 met the criteria for inclusion in the final analysis. Figure 1 shows the PRISMA flowchart for study selection. Included studies were published between 1991 and 2021, all studies were observational cohorts, and 6 were multicentric. One study was multinational, 4 originated from the United States, 2 from Canada, 2 from Japan, and 1 each from England, Netherland, Finland, Italy and China.

Tables 1, 2 shows the details of the included studies. Thirteen studies were based on risk-adjusted populations. A total of 17,942 patients were included in the final analysis. The number of patients in each study ranged from 128 to 10,141.

**Table 1:** Summary of included studies—part 1.

Author	Year of publication	Country	No of patients	Study design	Mean follow-up	Population comparability	Reported outcomes
Conrotto^20^	2014	Italy, Korea, Netherlands Lativa, USA, France, Germany	304 86 CABG 218 PCI	Retrospective, multicenter	3y	Propensity score matching	All-cause mortality Myocardial infraction Re-revascularization Stroke
Dacey^21^	2007	England	1,693 991 CABG 702 PCI	Retrospective, multicenter	8y	Cox proportional hazard regression	All-cause mortality Stroke
Garza^22^	2003	USA	239 128 CABG 111 PCI	Retrospective, single center	1.8y	Multivariant regression analysis	All-cause mortality Acute renal failure Myocardial infarction Stroke Re-revascularization
Gimbel^23^	2020	Netherlands	597 251 CABG 346 PCI	Retrospective, single center	4y	Cox proportional hazard regression	All-cause mortality Myocardial infarction Re-revascularization Stroke
Graham^24^	2002	Canada	983 133 CABG 289 PCI 561 OMT	Retrospective, multicenter	4y	Propensity score matching	All-cause mortality
Gunn^25^	2012	Finland	669 274 CABG 393 PCI	Retrospective, single center	3.6y	Propensity score matching	All-cause mortality
Hara^26^	2021	Japan	527 151 CABG 376 PCI	Retrospective, multicenter	5y	Cox proportional hazard regression	All-cause mortality Myocardial infarction Re-revascularization Stroke

*CABG* coronary artery bypass grafting, *PCI*  percutaneous coronary intervention, *OMT* optimal medical therapy.

**Table 2:** Summary of included studies—part 2.

Author	Year of publication	Country	No of patients	Study design	Mean follow-up	Population comparability	Reported outcomes
Kamiya^27^	2007	Japan	128 28 CABG 100 PCI	Retrospective, single center	5y	Cox proportional hazard regression	All-cause mortality
Kaul^28^	1994	USA	310 205 CABG 105 PCI	Retrospective, single center	8y	Cox proportional hazard regression	All-cause mortality Acute renal failure Stroke
Mick^29^	1991	USA	195 142 CABG 53 PCI	Retrospective, single center	3y	Not adjusted	All-cause mortality Myocardial infarction Re-revascularization Stroke Acute renal failure
Nicolini^30^	2015	Italy	1,388 441 CABG 947 PCI	Retrospective, multicenter	7y	Propensity score matching	All-cause mortality Myocardial infarction Stroke
Rodes-Cabau^31^	2008	Canada	249 145 CABG 104 PCI	Retrospective, single center	2y	Propensity score matching	All-cause mortality Myocardial infarction
Sheridan^32^	2010	USA	10,141 5803 CABG 4338 PCI	Retrospective, Multicenter	3y	Propensity score matching	All-cause mortality Myocardial infarction Re-revascularization Stroke
Wu^33^	2019	China	519 110 CABG 292 PCI 117 OMT	Retrospective, single center	2.1y	Multivariant regression analysis	Alll-cause mortality

*CABG* coronary artery bypass grafting, *PCI* percutaneous coronary intervention, *OMT* optimal medical therapy.

### Patient characteristics

Supplementary Table 3 summarizes the demographic data of the patient population in each study. Percentage of female patients ranged from 22.7 to 71.4%; percentage of mean LVEF ranged from 48 to 60.6%; percentage of hypertension ranged from 26 to 90%; percentage of diabetes ranged from 14.6 to 93%; percentage of positive smoking status ranged from 3 to 34.9%; percentage of prior CVA ranged from 4.2 to 17.4%; percentage of prior MI ranged from 17.8 to 67.1%; percentage of prior PCI ranged from 7.8 to 17.4%; percentage of renal failure ranged from 1 to 13.5% and the percentage of COPD ranged from 2 to 18.9%. Supplementary Table 3 shows the specific description of the included outcomes.

### Meta-analysis

**Figure 2:**
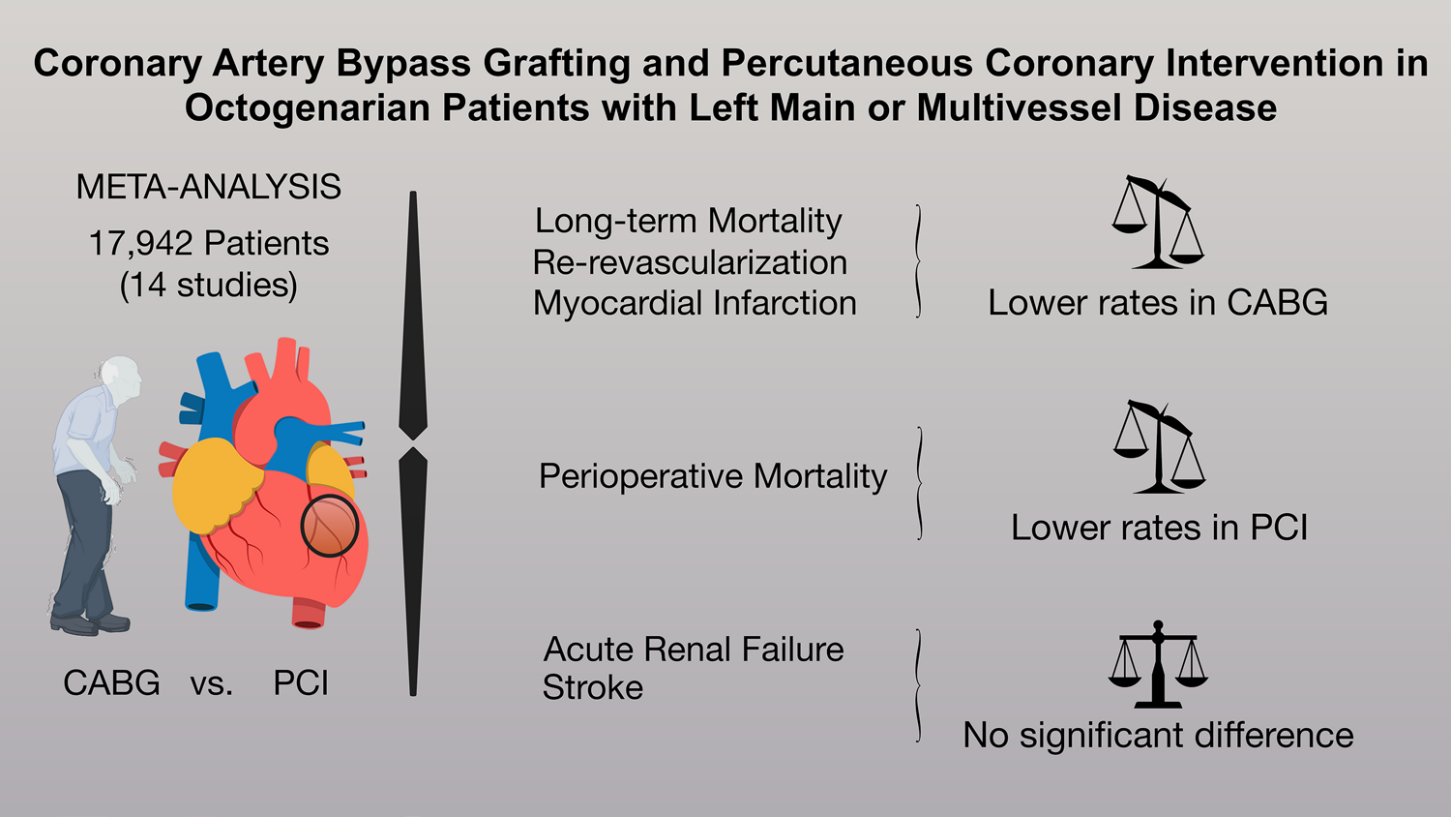
(Central Picture). Graphical abstract showing the main findings of the analysis.

Figure 2 and Table 3 outline the detailed results of the meta-analysis.

**Table 3:** Outcomes summary.

Outcome	Number of studies	Number of patients	Effect estimate (95%CI, p-value)
Long-term all-cause mortality	12	15,461	HR = 1.23, 1.05–1.44, p < 0.01
Perioperative all-cause mortality	7	12,454	RR = 1.21, 1.07–1.36, p < 0.01
Acute renal failure	3	744	RR = 1.21, 0.54–2.72, p = 0.64
Myocardial infarction	8	12,844	RR = 0.51, 0.46–0.56, p < 0.01
Re-revascularization	6	12,003	RR = 0.31, 0.22–0.45, p < 0.01
Stroke	9	14,598	RR = 1.52, 0.96–2.39, p = 0.07

*CI* confidence interval, *HR* hazard ratio, *RR* relative risk.

### Primary outcome

**Figure 3:**
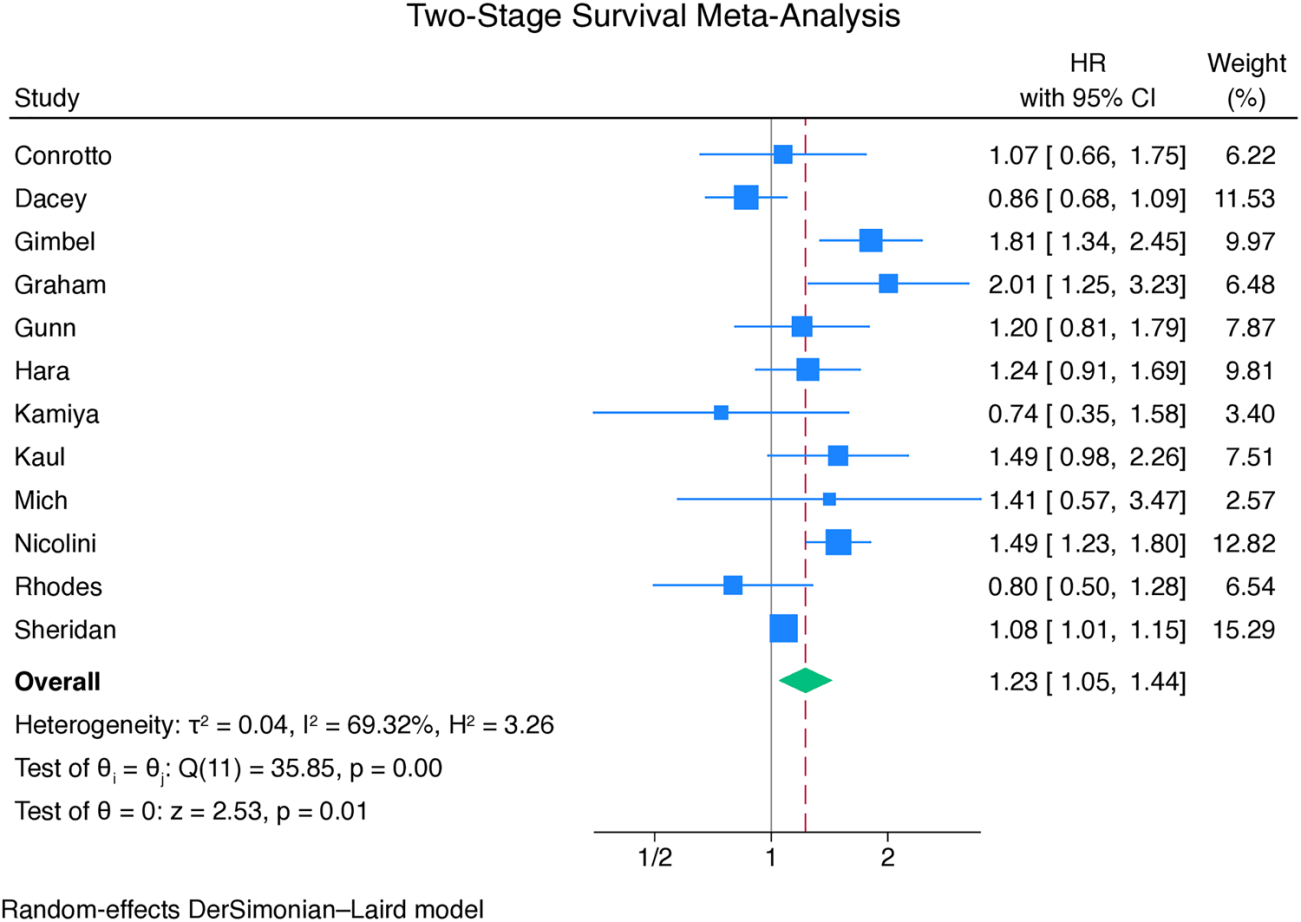
Forest plot for long-term all-cause mortality. *CI* confidence interval, *HR* hazard ratio.

Figure 3 shows the forest plot for long-term all-cause mortality. The patients who underwent CABG showed lower incidence of long-term all-cause mortality (HR = 1.23, 95% CI 1.05–1.44, p < 0.01).

Supplementary Fig. 2 shows the leave-one-out analysis showing that most of the studies confirm the robustness of the analysis, with minimal variations of the confidence interval. Supplementary Fig. 3 provides the funnel plot for the publication bias assessment.

Supplementary Fig. 4 shows a sub-group analysis dividing studies according to the publication year. There was no significant difference between the two therapy groups in studies published before and after 2010 (p-interaction = 0.49).

### Individual patient data and survival curve reconstruction

Overall, 12 Kaplan–Meier curves were processed, digitalized, and reconstructed. A side-by-side comparison of our reconstructed Kaplan–Meier curves and those found in the original publications is provided in Supplementary Fig. 5. Using the previously described methodology, we extracted the IPD from these curves.

### Overall survival analysis

**Figure 4:**
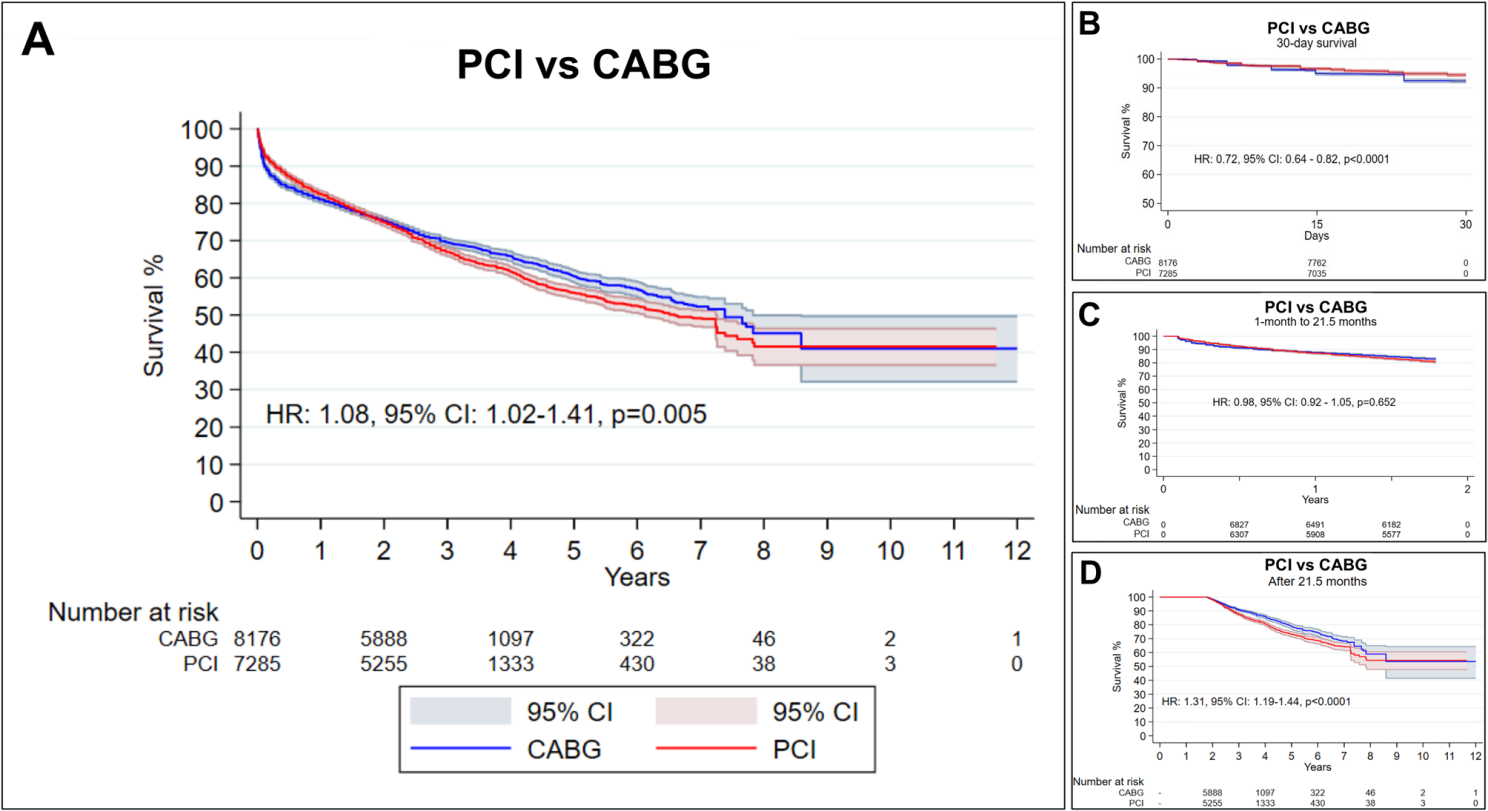
Pooled Kaplan–Meier curves showing the cumulative risk of all-cause mortality following CABG and PCI. *CI* confidence interval, *HR* hazard ratio.

Figure 4 shows the pooled Kaplan–Meier curves of reconstructed IPD. Patients who underwent CABG had significantly lower risk of death in the follow-up compared to those who underwent PCI (HR: 1.08, 95% CI 1.02–1.41, *p* = 0.005).

Violation of the proportional hazards assumption was observed between scaled Schoenfeld residuals and follow-up time, as well as in log–log survival plots (Supplementary Fig. 6). This indicated that the HR is not constant over time.

Since we observed that the proportional hazards assumption was violated, we proceeded with landmark analysis, designating 21.5 months (the point where both curves crossed in Fig. 4A) as the landmark timepoint.

Figure 4B shows the 30-day survival analysis, which suggested that PCI offers a survival advantage compared to CABG (HR: 0.72, 0.64–0.82, p < 0.0001).

Figure 4C shows the landmark analysis from 1 to 21.5 months, which suggested that the two techniques offer comparable survival outcomes compared (HR: 0.98, 95% CI 0.92–1.05, *p* = 0.652).

Figure 4D shows the landmark analysis after 21.5 months of follow-up, which revealed a statistically significant survival advantage in favor of CABG over PCI (HR: 1.31, 95% CI 1.19–1.44, *p* < 0.0001).

### Secondary outcomes

Supplementary Fig. 7 shows the forest plot for perioperative all-cause mortality. The patients who underwent CABG showed higher incidence of perioperative all-cause mortality (RR 1.21, 95%CI 1.07–1.36, p < 0.01).

Supplementary Fig. 8 shows the forest plot for acute renal failure. There was no significant difference between the two therapy groups (RR 1.21, 95%CI 0.54–2.72, p = 0.64).

**Figure 5:**
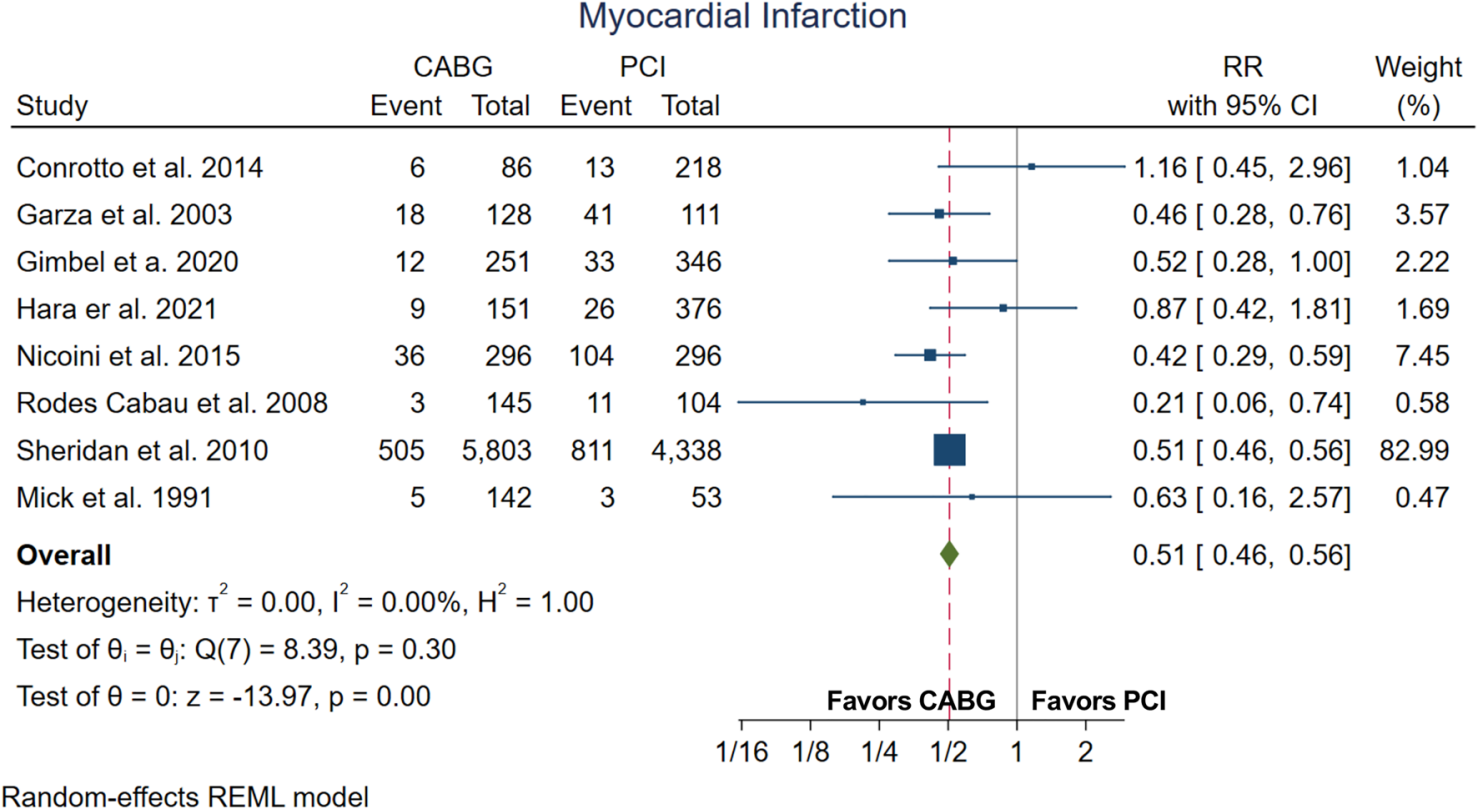
Forest plot for myocardial infarction. *CI* confidence interval, *RR* relative risk.

Figure 5 shows the forest plot for myocardial infarction. The patients who underwent CABG showed lower incidence of myocardial infarction (RR 0.51, 95%CI 0.46–0.56, p < 0.01).

Supplementary Fig. 9 shows the forest plot for re-revascularization. The patients who underwent CABG showed lower incidence of re-revascularization (RR 0.31, 95%CI 0.22–0.45, p < 0.01).

Supplementary Fig. 10 shows the forest plot for stroke. There was no significant difference between the two therapy groups (RR 1.52, 95%CI 0.96–2.39, p = 0.07).

## Discussion

Our analysis suggests that for the treatment of octogenarian patients with left main or multivessel disease CABG is associated with significantly lower risk of death in the long-term follow-up compared to PCI (survival advantage after the first 21.5 months of follow-up). This superiority of CABG is associated with fewer events of myocardial infarction and re-revascularization. However, perioperative mortality is higher with CABG.

Our results are relevant as they provide valuable information, affecting the treatment of a substantial number of patients. Currently, there are aproximately 147 milion octogenarians worldwide, representing 1.9% of the global population^8^. This global number of octagenarians has risen significantly over the last decades and is projected to rise further. As age is the strongest factor connected with the development of coronary artery disease (CAD)^9^, this relates in millions of patients worldwide faced with a with a potential treatment choice between CABG or PCI. Necropsy studies for example, have demonstrated a high prevalence (∼ 60%) of obstructive CAD in patients ≥ 80 years of age, often with features of advanced disease [e.g., calcification (80% to 90%), multivessel disease (40%)]^9,10^. The prevalence of CAD (defined as coronary insufficiency, myocardial infarction, angina pectoris, or CAD-related death) in octogenarins was reported with 31.2% for the age group 85–89 by the Franmingham Heart Study and similar rates were observed by the Cardiovascular Health Study (30.9% for 80–84 years and 35.8% for 85–89 years)^10^.

In this context, one of the main findings of our work is the fact that the survival curves of CABG and PCI in octogenarian patients cross (similar to other known studies comparing CABG and PCI) after approximately 2 years.

Currently treatment recommendations and guideliens for CAD outlining when to recommend which treatment option (e.g., CABG or PCI) are mostly based on randomized trials, which are bound to reflect the average treatment effect for an often selected patient population^11^. Despite the huge number of octagenarians affected by CAD worldwide, they have been typically under-represented in randomized cardiovascular clinical trials as many studies have either excluded older patients or only included those at lower risk^12^. For example, the mean age of the randomized patients in the EXEL trial was 66 years^13^ and and 65 years in the FAME 3 clinical trial^14^. Thus our work may fill the evidence gap resulting from the underrepresentation of octogenarian patients in such trials, as it summarizes the current data and presents information not provided by randomized trials.

However, it is so far unknown if quality of life measures, functional status, and overall well-being also follow this trend. Understanding how these factors are affected by CABG or by PCI in octogenarians can provide valuable insights for personalized treatment recommendations and should be a matter of future research.

Our results, once again put center stage the question of the underlying mechanisms leading to and explaining them. As mentioned earlier, the concept of surgical collateralization might explain the life-prolonging (i.e., prognostic) effect of CABG, which appears to be due to prevention of future myocardial infarctions^1^. Our work once more confirms this concept, as the survival advantage for CABG was as expected associated with fewer events of myocardial infarctions and re-revascularization. This infarct-preventing mechanism through CABG collateralization might be also the explanation, why the long-term survival of elderly patients after CABG is superior to that of their age-matched population^15,16^.

CABG has always been considered the more invasive treatment option compared to PCI, and in octogenarian patients physicians have often been reluctant to recommend it, especially in cases with other comorbidities. However, in our analysis we did not find any significant difference in the rates of acute kidney injury and stroke between the groups. Furthermore, recent data has illustrated that generally CABG might be the superior treatment in patients with diabetes^17^ or chronic kidney disease^18^ and/or dialysis^19^. Thus, it seems that the treatment benefit of CABG does not diminish in patients of advanced age and/or comorbidities, and our results support that probability.

One of the main findings of our work is the fact that the survival curves of CABG and PCI in octogenarian patients cross (similar to other known studies comparing CABG and PCI) after approximately 2 years. However, it is so far unknown if quality of life measures, functional status, and overall well-being also follow this trend. Understanding how these factors are affected by CABG or by PCI in octogenarians can provide valuable insights for personalized treatment recommendations and should be a matter of future research.

Nevertheless, this information is essential as it may enable individualized approach in octogenarians- ones with clearly limited life expectancy might be be mor suiutable for PCI, but octogenarians with longer life expectancy should be informed about this trade-off between initial risk and survival advantage.

### Study strength and limitations

This is the first meta-analysis of reconstructed time-to-event data to address this important topic. Moreover, we analyzed 4 different outcomes besides mortality. However, this work has the intrinsic limitations of observational series, including the risk of methodological heterogeneity of the included studies and residual confounders. In addition, treatment allocation bias is likely present in all observational series comparing two therapies with different operative risk and invasiveness. Moreover, one study contributed significantly for the final sample size, which could contribute strongly for the final treatment effect.

## Conclusion

The results support the concept that CABG provides a survival advantage over PCI for complex CAD over time even in octogenarians. This survival advantage is associated with fewer events of myocardial infarction and re-revascularization; however, it comes with an increased risk in the perioperative mortality after CABG. Since survival curves cross after approximately 2 years, octogenarians with longer life expectancy should be informed about this trade-off between initial risk and survival advantage.

### Supplementary Information

Supplementary Information.

## Data Availability

The data underlying this article are available in the article and in its online supplementary material.
