# Supplementary material for: Comparing outcomes between coronary artery bypass grafting and percutaneous coronary intervention in octogenarians with left main or multivessel disease
Source: Sci Rep. 2023 Dec 15;13:22323. doi: 10.1038/s41598-023-49069-2 (PMC10724226; doi:10.1038/s41598-023-49069-2)
Supplement: Supplementary file 1 — Supplementary Information. [file 41598_2023_49069_MOESM1_ESM.docx]

**Supplementary Material**

**Supplementary Table 1.** Search strategy for Ovid MEDLINE.

**Supplementary Table 2.** Demographics of included patients from the selected studies.

**Supplementary Table 3.** Definition of the outcomes of interest by study.

**Supplementary Figure 1.** The Risk of Bias in Non-Randomized Studies of Interventions tool (ROBINS-I) diagrams.

**Supplementary Figure 2.** Leave-one-out analysis for long-term all-cause mortality.

**Supplementary Figure 3.** Funnel plot for assessment of publication bias for the primary outcome (long-term all-cause mortality).

**Supplementary Figure 4.** Sub-group analysis dividing studies according to the publication year for long-term mortality.

**Supplementary Figure 5.** Side-by-side comparison of our reconstructed Kaplan–Meier curves and the original ones.

**Supplementary Figure 6.** Test of proportional hazard assumption (A) and in log–log survival plots (B).

**Supplementary Figure 7.** Forest plot for perioperative all-cause mortality.

**Supplementary Figure 8.** Forest plot for acute renal failure.

**Supplementary Figure 9.** Forest plot for re-revascularization.

**Supplementary Figure 10.** Forest plot for stroke.

**Supplementary Table 1.** Search strategy for Ovid MEDLINE.

| Search: **(octogenarians) AND (coronary artery bypass grafting) AND (percutaneous coronary intervention)**  ("octogenarians"[MeSH Terms] OR "octogenarians"[All Fields] OR "octogenarian"[All Fields]) AND ("coronary artery bypass"[MeSH Terms] OR ("coronary"[All Fields] AND "artery"[All Fields] AND "bypass"[All Fields]) OR "coronary artery bypass"[All Fields] OR ("coronary"[All Fields] AND "artery"[All Fields] AND "bypass"[All Fields] AND "grafting"[All Fields]) OR "coronary artery bypass grafting"[All Fields]) AND ("percutaneous coronary intervention"[MeSH Terms] OR ("percutaneous"[All Fields] AND "coronary"[All Fields] AND "intervention"[All Fields]) OR "percutaneous coronary intervention"[All Fields]) |
| --- |
| **Translations** |
| **octogenarians:** "octogenarians"[MeSH Terms] OR "octogenarians"[All Fields] OR "octogenarian"[All Fields]  **coronary artery bypass grafting:** "coronary artery bypass"[MeSH Terms] OR ("coronary"[All Fields] AND "artery"[All Fields] AND "bypass"[All Fields]) OR "coronary artery bypass"[All Fields] OR ("coronary"[All Fields] AND "artery"[All Fields] AND "bypass"[All Fields] AND "grafting"[All Fields]) OR "coronary artery bypass grafting"[All Fields]  **percutaneous coronary intervention:** "percutaneous coronary intervention"[MeSH Terms] OR ("percutaneous"[All Fields] AND "coronary"[All Fields] AND "intervention"[All Fields]) OR "percutaneous coronary intervention"[All Fields] |

**Supplementary Table 2.** Demographics of included patients from the selected studies

| Study | Age  (mean±SD) | | Female (%) | | LVEF (Mean±SD) | | HP (%) | | DM (%) | | Smoking (%) | | Prior CVA (%) | | | Prior MI (%) | | | Prior PCI (%) | | | Renal failure (%) | | | COPD (%) | |  |
| --- | --- | --- | --- | --- | --- | --- | --- | --- | --- | --- | --- | --- | --- | --- | --- | --- | --- | --- | --- | --- | --- | --- | --- | --- | --- | --- | --- |
|  | **CABG** | **PCI** | **CABG** | **PCI** | **CABG** | **PCI** | **CABG** | **PCI** | **CABG** | **PCI** | **CABG** | **PCI** | **CABG** | **PCI** | **CABG** | | **PCI** | **CABG** | | **PCI** | **CABG** | | **PCI** | **CABG** | | **PCI** |  |
| Garza | 81.9±1.9 | 83.5±2.8 | 42 | 49 | NR | NR | NR | NR | NR | NR | NR | NR | NR | NR | NR | | NR | NR | | NR | NR | | NR | NR | | NR |  |
| Darcey | 82.8 | 83.1 | 45.3 | 56.7 | NR | NR | NR | NR | 24.5 | 22.9 | NR | NR | NR | NR | 48.7 | | 53.5 | NR | | NR | 4.6 | | 9.1 | 10.4 | | 10.5 |  |
| Gimbel | 79±3.4 | 80±3.9 | 27 | 35 | NR | NR | NR | NR | 75 | 93 | NR | NR | NR | NR | NR | | NR | NR | | NR | 2.4 | | 3.2 | NR | | NR |  |
| Conrotto | 83±2.9 | 83.6±3.2 | 45.3 | 35.3 | 53.3±11.4 | 49.8±14.3 | 74.1 | 76.6 | 23.2 | 26.1 | 29.1 | 29.4 | NR | NR | NR | | NR | 8.1 | | 17.4 | 7 | | 11 | NR | | NR |  |
| Graham | 81 | 81.9 | 26.1 | 44.6 | NR | NR | 59.5 | 55.7 | 15.8 | 18 | NR | NR | 12 | 11.1 | 57.1 | | 67.1 | 11.3 | | 13.8 | 3 | | 3.8 | NR | | NR |  |
| Gunn | 82±1.7 | 83.3±2.5 | 39 | 49 | NR | NR | 68 | 79 | 18 | 22 | NR | NR | 6 | 6 | 56 | | 77 | 9 | | 11 | NR | | NR | NR | | NR |  |
| Hara | 82 | 83 | 36 | 44 | NR | NR | 85 | 90 | 40 | 34 | 13 | 13 | 11 | 20 | 33 | | 23 | NR | | NR | 1 | | 13 | 2 | | 3.7 |  |
| Kamiya | 82±2.1 | 83±2.8 | 50 | 37 | 57±18 | 52±16 | 57.1 | 60 | 28.6 | 40 | NR | NR | NR | NR | NR | | NR | NR | | NR | NR | | NR | NR | | NR |  |
| Kaul | 82.4±2 | 84±3 | 45.8 | 71.4 | NR | NR | 26.3 | 26 | 14.6 | 17.8 | 20.5 | 31.25 | NR | NR | NR | | NR | 7.8 | | 4.2 | NR | | NR | 15.6 | | 11.4 |  |
| Mick | 82±2 | 82±2 | 39 | 47 | NR | NR | 53 | 58 | 18 | 21 | 41 | 32 | NR | NR | 49 | | 43 | NR | | NR | NR | | NR | NR | | NR |  |
| Nicolini | NR | NR | 37.5 | 39.54 | NR | NR | 84.1 | 83.1 | 20.3 | 19.6 | 5.4 | 6.1 | 19.3 | 16.9 | 43.6 | | 38.9 | NR | | NR | 11.5 | | 13.5 | 7.9 | | 8.1 |  |
| Rodes Cabau | 82±2 | 85±3 | 37 | 46 | 56±14 | 48±15 | 72 | 75 | 26 | 27 | 6 | 3 | 16 | 16 | 59 | | 49 | 8 | | 14 | 1 | | 8 | 15 | | 20 |  |
| Sheridan | 86.6 | 87.7 | 45.1 | 58.1 | NR | NR | 71.2 | 75.2 | 21.3 | 22 | NR | NR | 5.7 | 5.9 | 17.8 | | 24.3 | NR | | NR | NR | | NR | 18.9 | | 17.8 |  |
| Wu | 81.2±1.7 | 81.5±1.9 | 22.7 | 34.6 | 59.9±8.4 | 60.6±9.8 | 72.7 | 68.2 | 33.6 | 34.9 | 30.9 | 34.9 | NR | NR | NR | | NR | NR | | NR | NR | | NR | NR | |  |  |

CABG= coronary arterial bypass graft; CVA= cerebrovascular accident; DM= diabetes; FEM= female; HP= hypertension; LVEF= left ventricular ejection fraction; MI= myocardial infarction; NR= not reported; PCI= percutaneous coronary intervention; SD= standard deviation.

**Supplementary Table 3.** Definition of the outcomes of interest by study.

| Author | Acute kidney injury | Myocardial infarction |
| --- | --- | --- |
| Garza | Increase in serum creatinine 0.5 mg/dl above the baseline preprocedural value | Myocardial infarction (MI) was classified into ST-segment elevation MI (2 contiguous leads with 1-mm ST-segment elevation on a 12-lead electrocardiogram) or non–ST-segment elevation MI, defined as an increase of creatine kinase-MB 3 times the upper limit of normal |
| Gimbel | Outcome not included | Fourth Universal Definition of myocardial infarction |
| Conrotto | Outcome not included | Spontaneous MI was defined as the occurrence after hospital discharge of any value of troponin and/or CK-myocardial band greater than the upper limit of normal if associated with clinical and/or electrocardiographic change |
| Hara | Outcome not included | Arterial Revascularization Therapy (ART) definition |
| Nicolini | Outcome not included | Any hospital admission occurring after the index procedure with a principal diagnosis of myocardial infarction |
| Rodes Cabau | Outcome not included | Periprocedural MI (within the 7 days after intervention) was defined as an elevation of serum creatine kinase-MB isoenzyme that was 5 times the upper limit of normal after CABG and 3 times the upper limit of normal after PCI. In case of elevated creatine kinase-MB levels at baseline, MI was defined as an increase 50% over baseline values after intervention in both groups. MI after the periprocedural period was defined as any typical rise above the upper range limit and fall of biochemical markers of myocardial necrosis (either creatine kinase-MB or troponin) with at least one of the following: cardiac symptoms, development of Q waves on the ECG, or ECG changes indicative of ischemia |

MI= myocardial infarction.

**Supplementary Figure 1.** The Risk of Bias in Non-Randomized Studies of Interventions tool (ROBINS-I) diagrams.

**
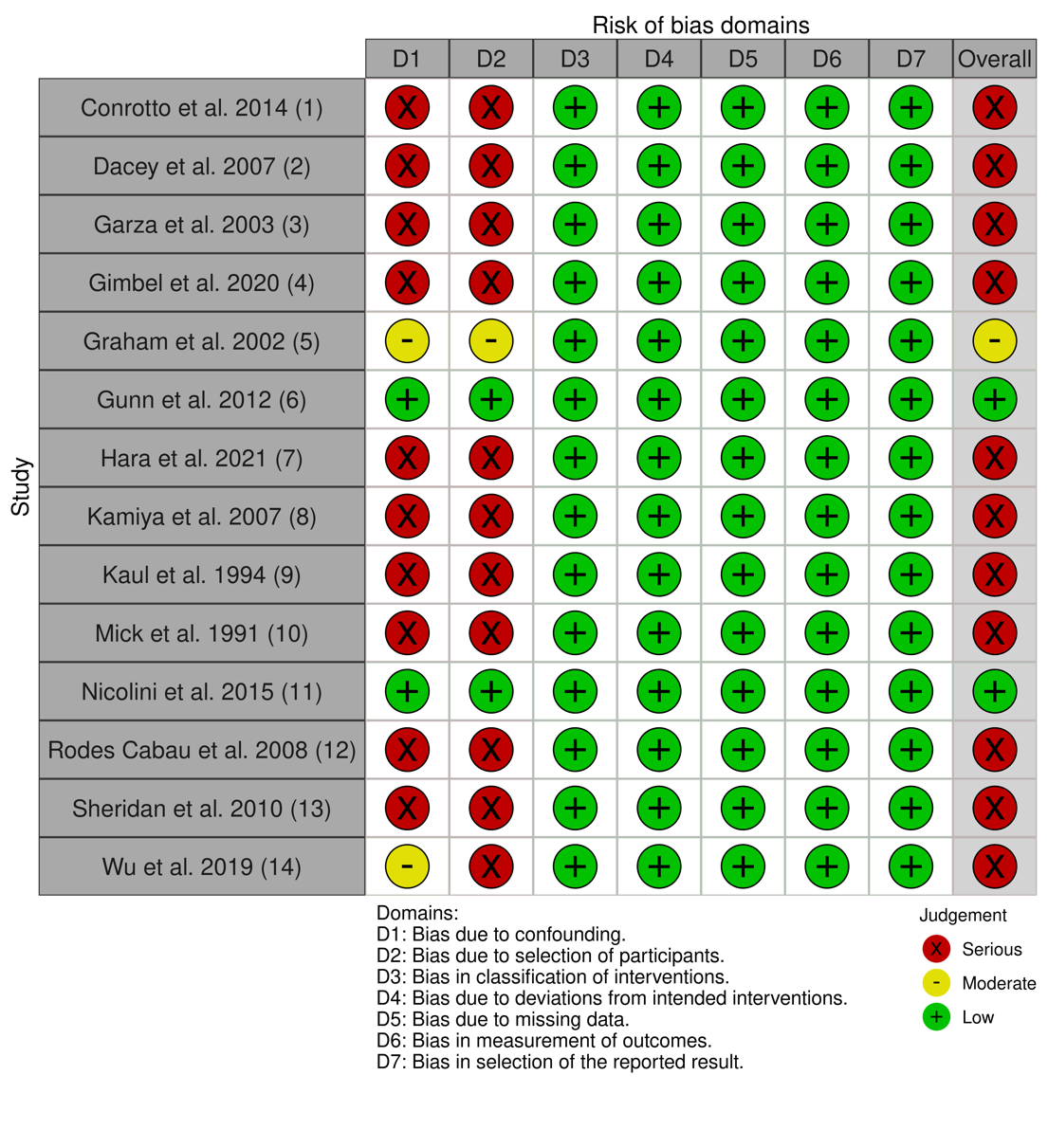
**

**
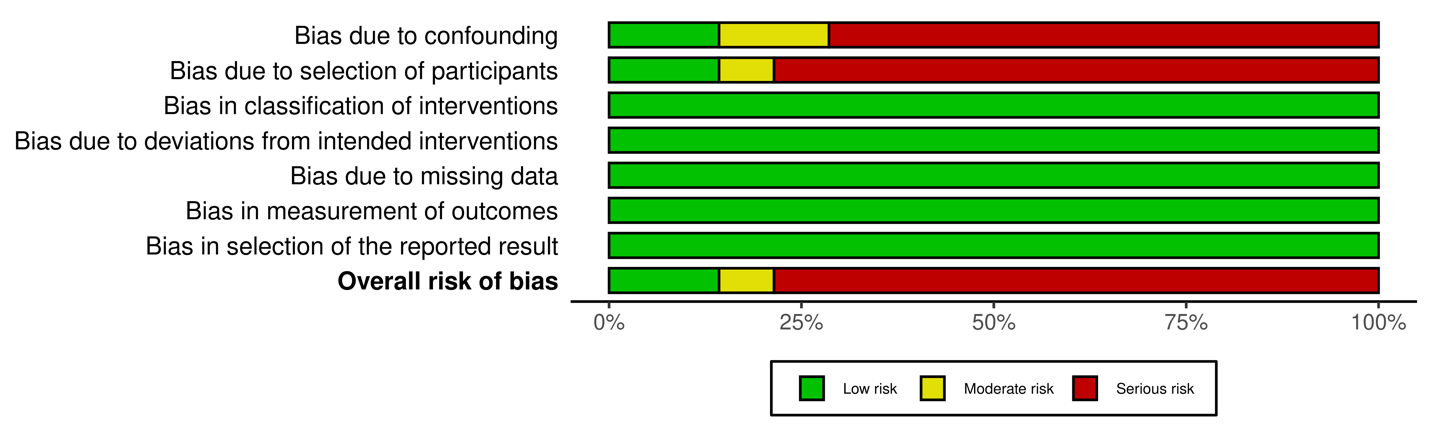
**

**Supplementary Figure 2.** Leave-one-out analysis for long-term all-cause mortality.


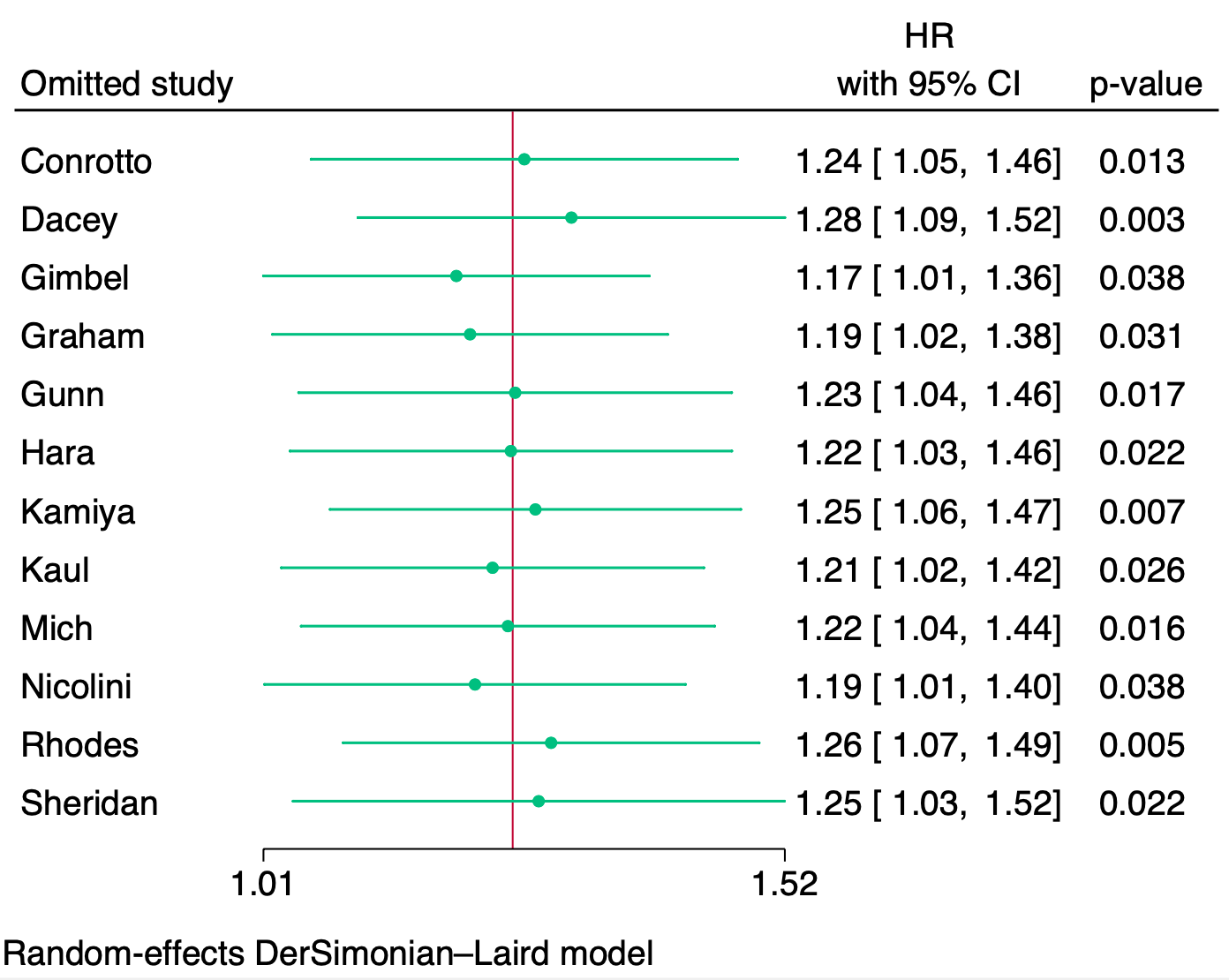


**Supplementary Figure 3.** Funnel plot for assessment of publication bias for the primary outcome (long-term all-cause mortality).


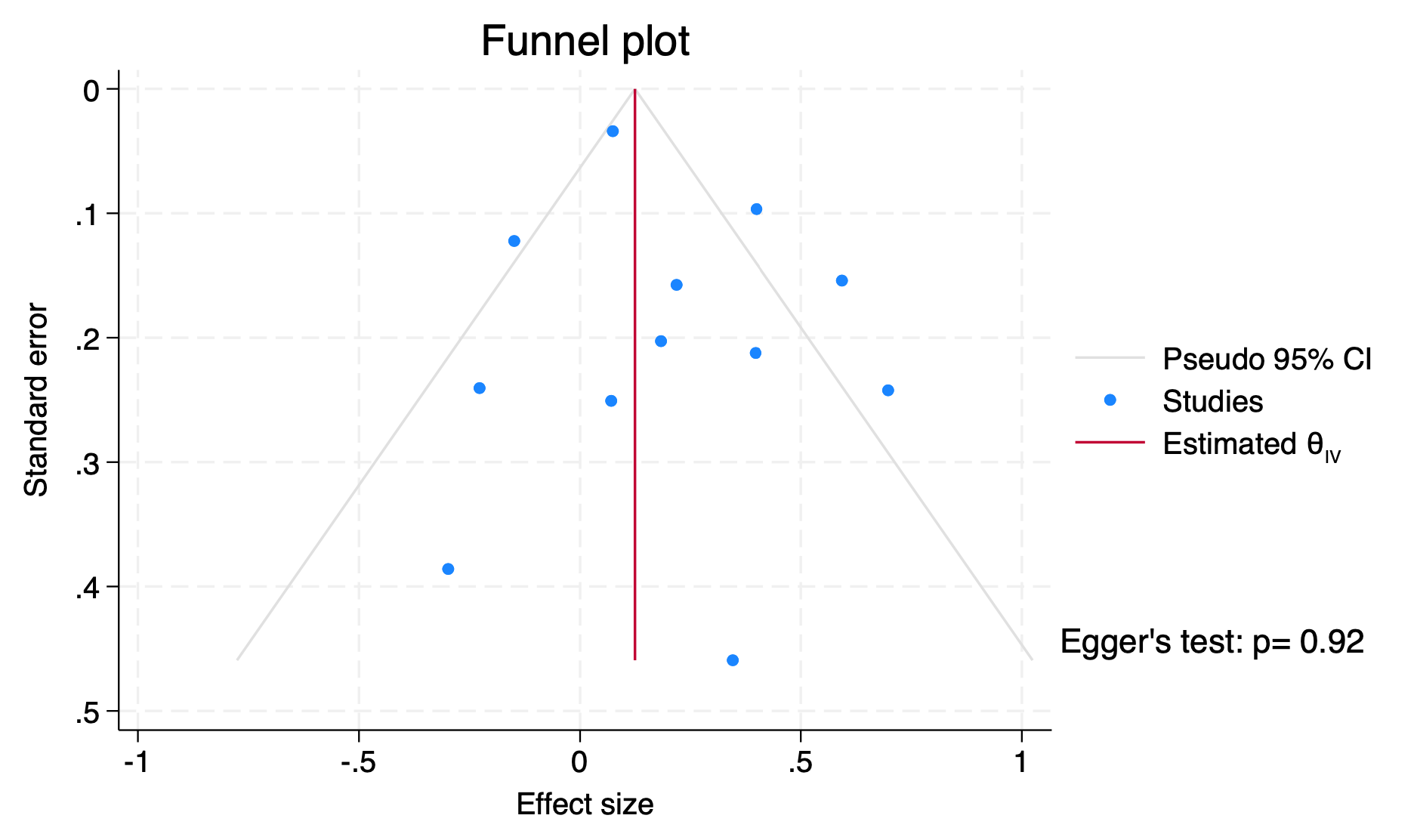


**Supplementary Figure 4.** Sub-group analysis dividing studies according to the publication year for long-term mortality.


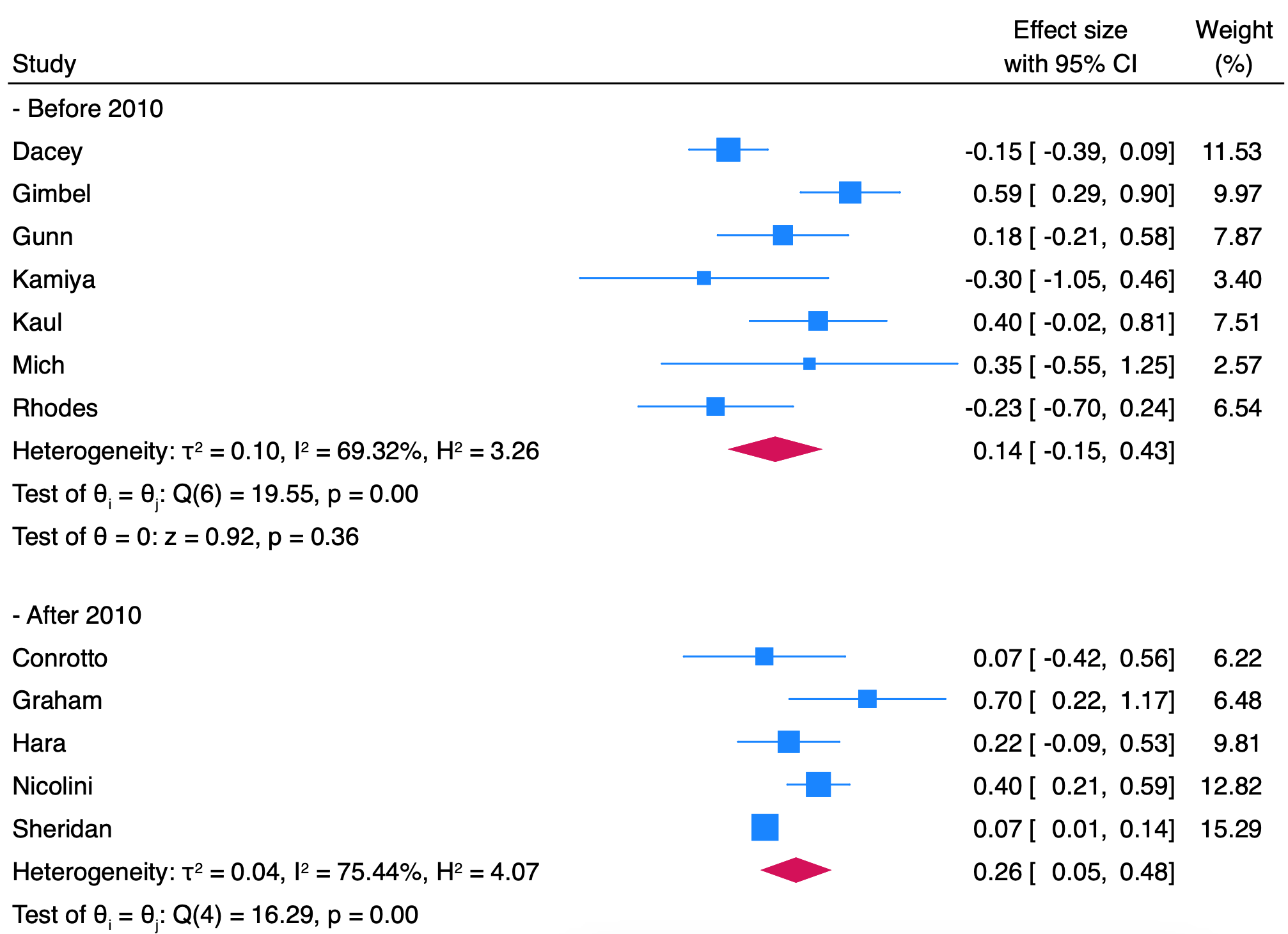


**Supplementary Figure 5.** Original Kaplan-Meier curves processed for the digitalization of the pooled curve (part 1).

A – Conrotto et al, 2014


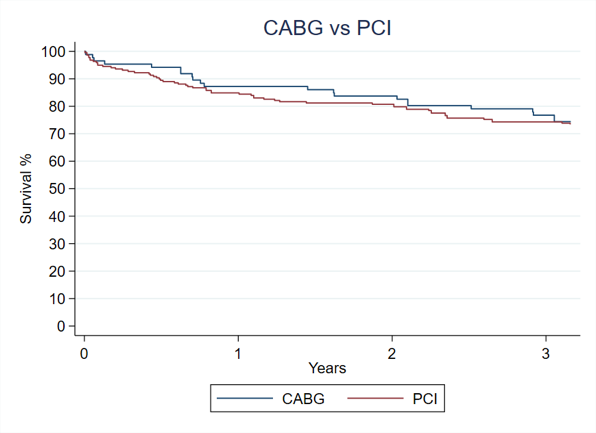

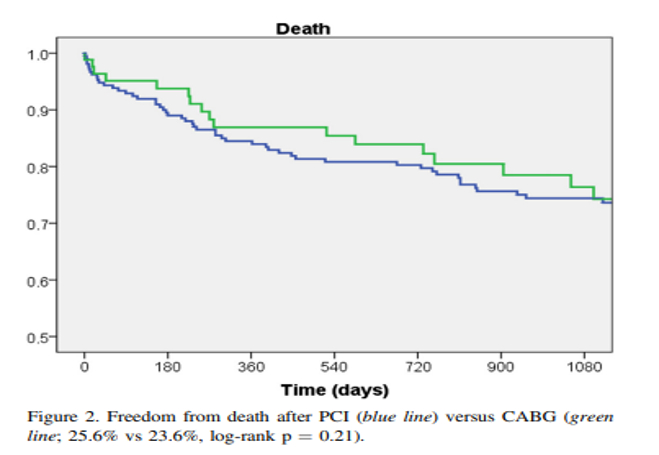


B – Dacey et al, 2007


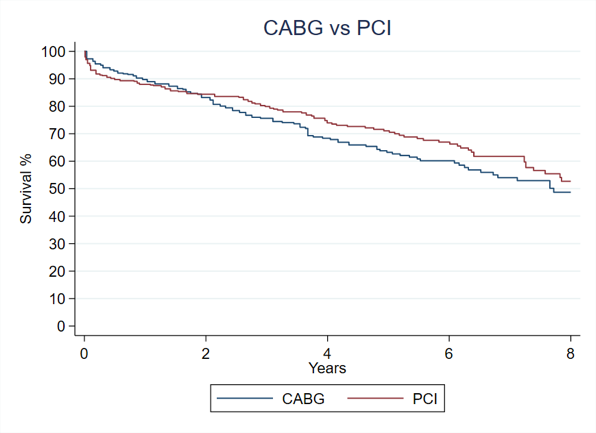

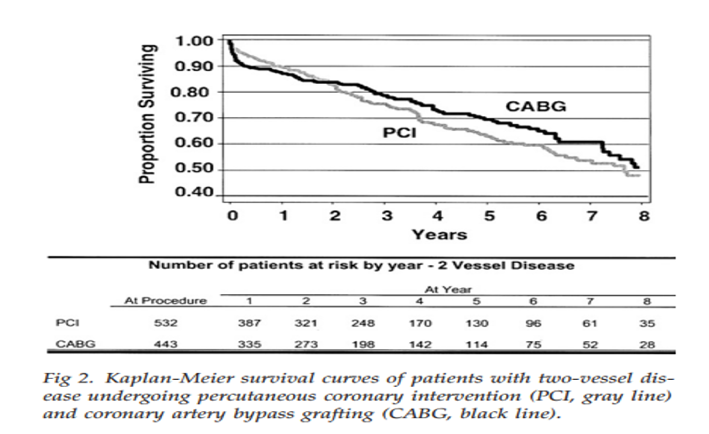


C – Gimbel et al, 2020


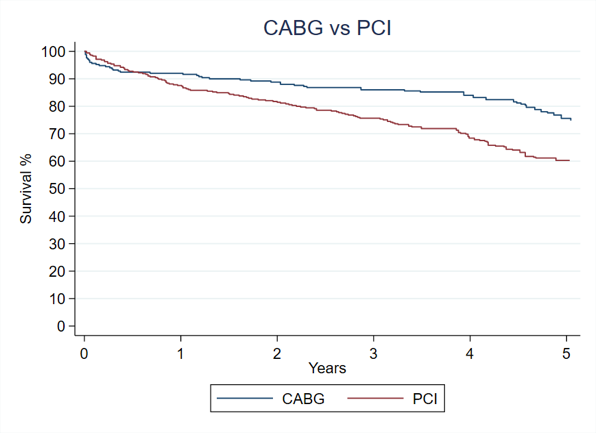

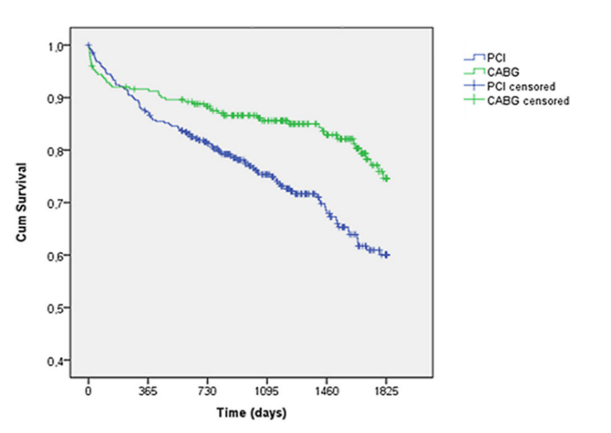


**Supplementary Figure 5.** Original Kaplan-Meier curves processed for the digitalization of the pooled curve (part 2).

D – Graham et al, 2002


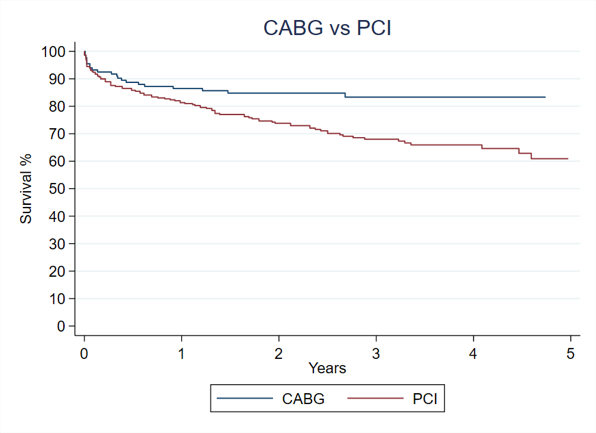

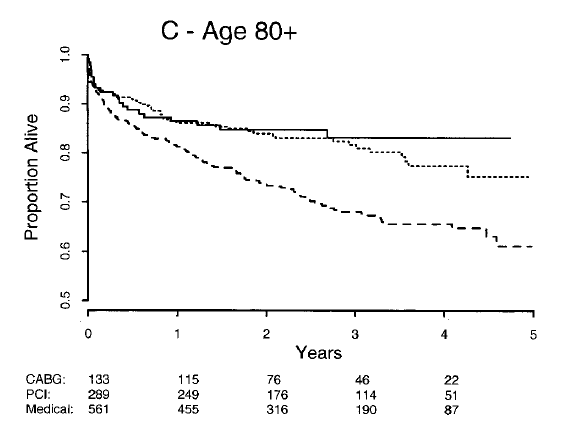


E – Gunn et al, 2012


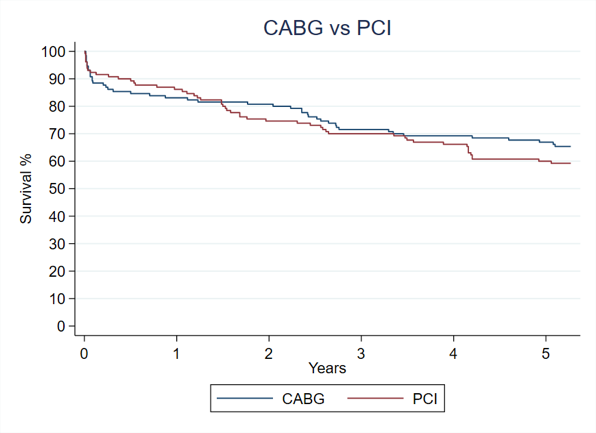

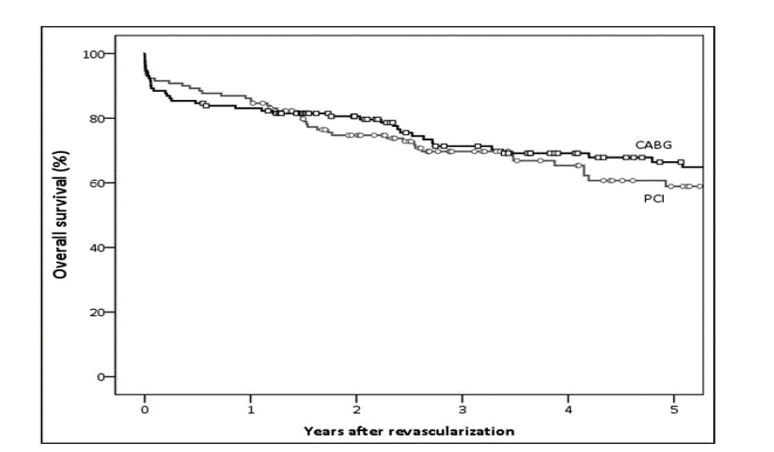


F – Hara et al, 2002


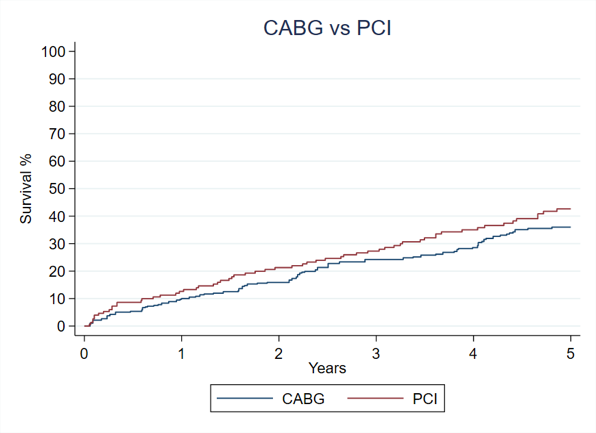

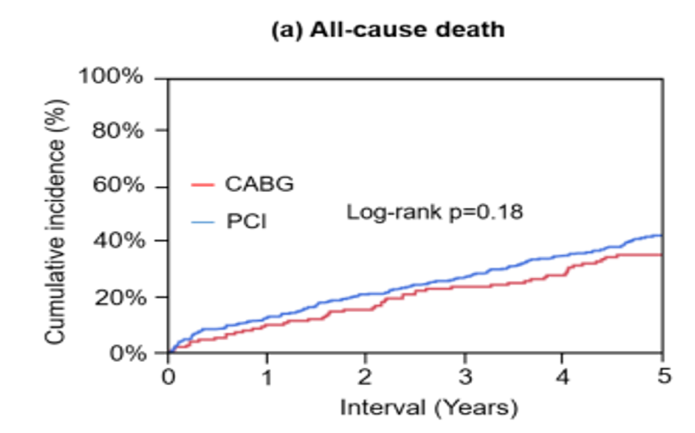


**Supplementary Figure 5.** Original Kaplan-Meier curves processed for the digitalization of the pooled curve (part 3).

G – Kamiya et al, 2007


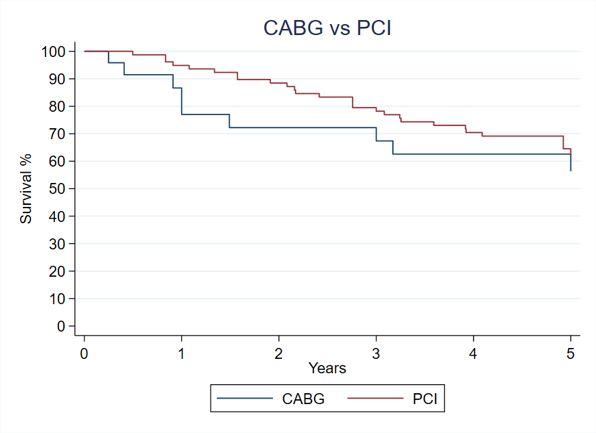

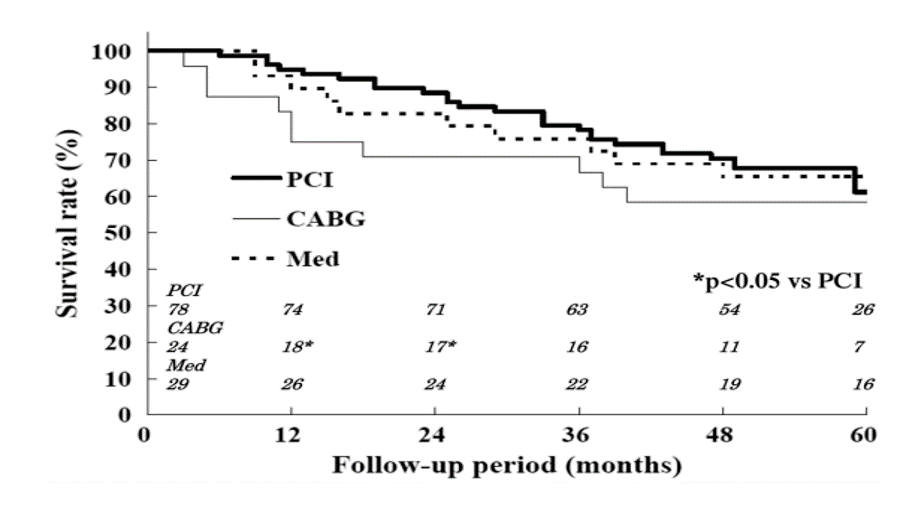


H – Kaul et al, 1994


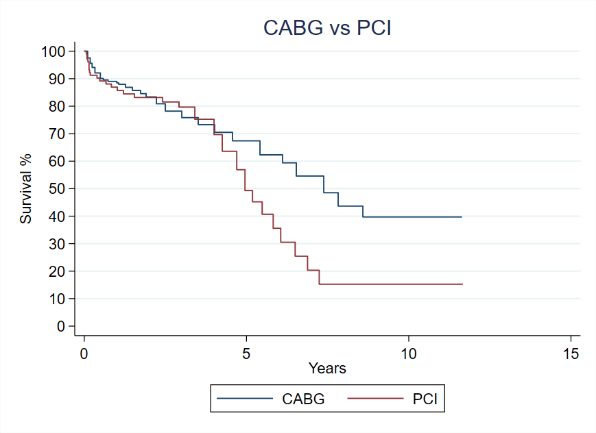

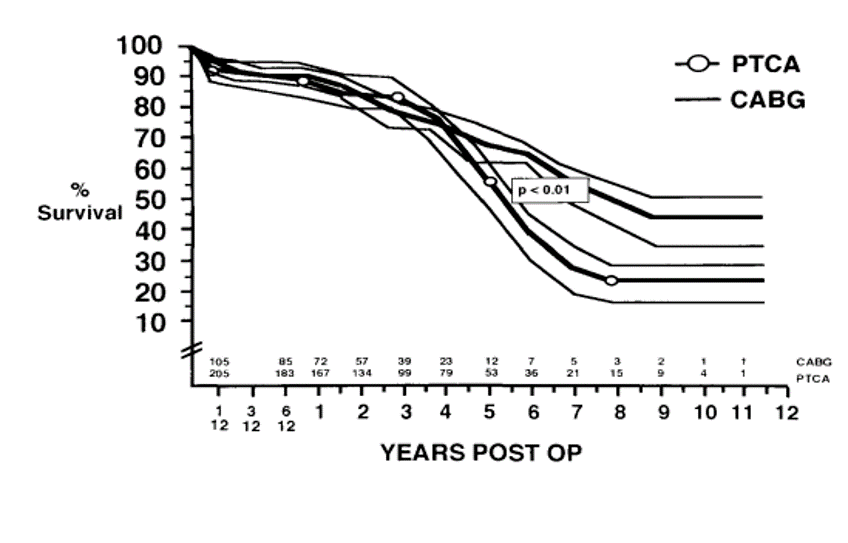


I – Mick et al, 1991


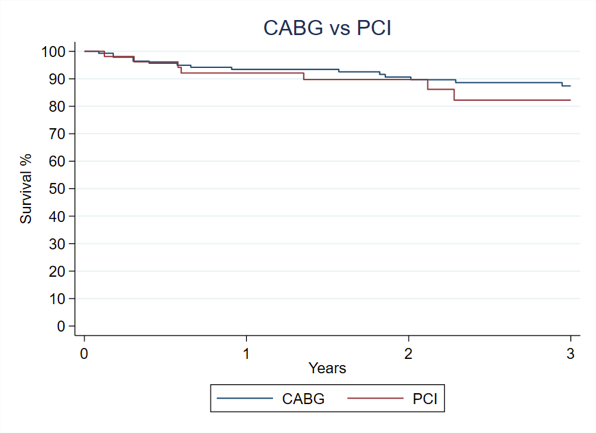

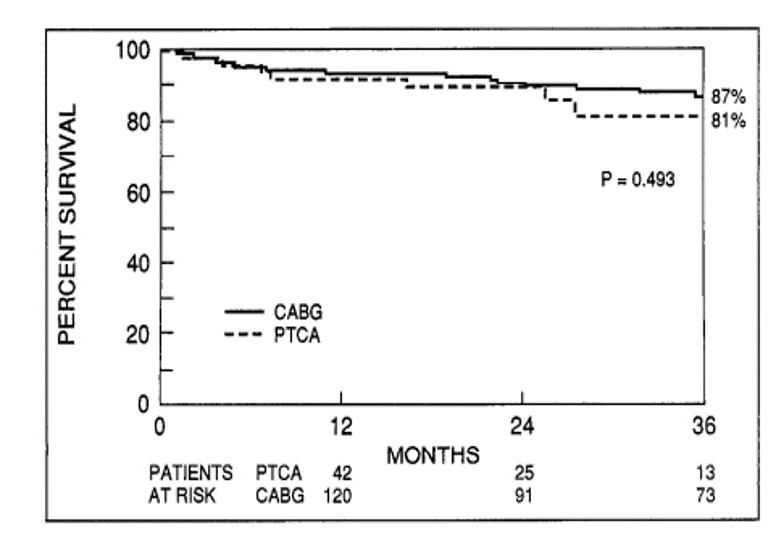


**Supplementary Figure 5.** Original Kaplan-Meier curves processed for the digitalization of the pooled curve (part 4).

J – Nicolini et al, 2015


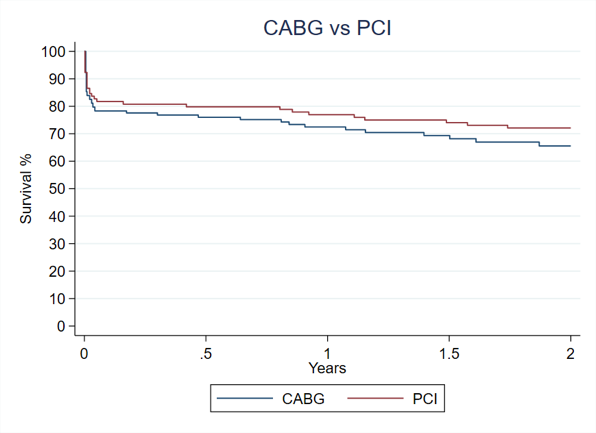

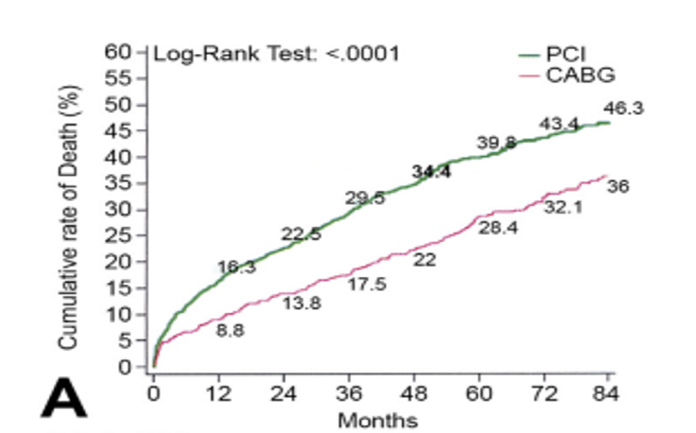


K – Rodes-Cabau et al, 2008


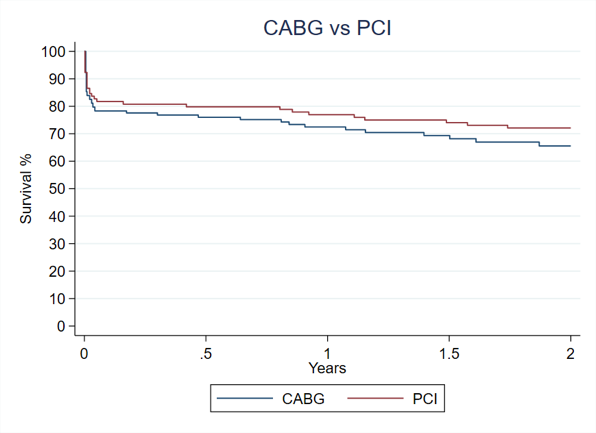

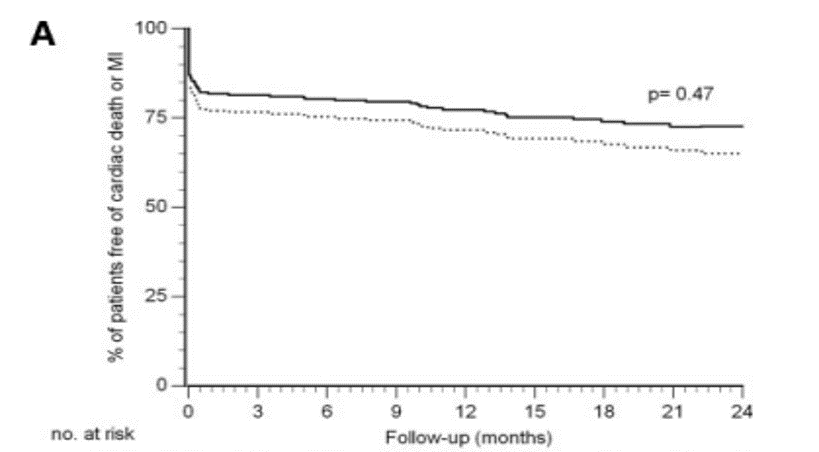


L – Sheridan et al, 2010


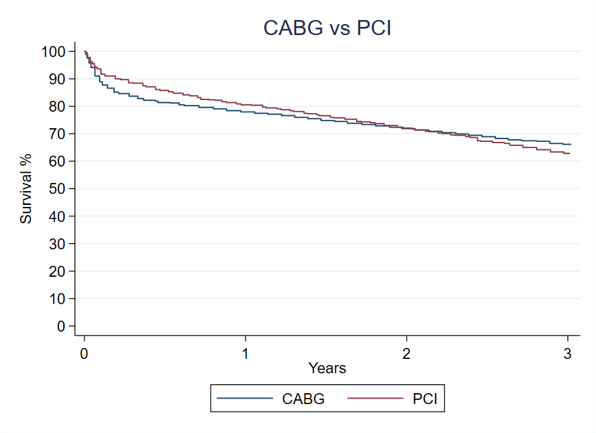

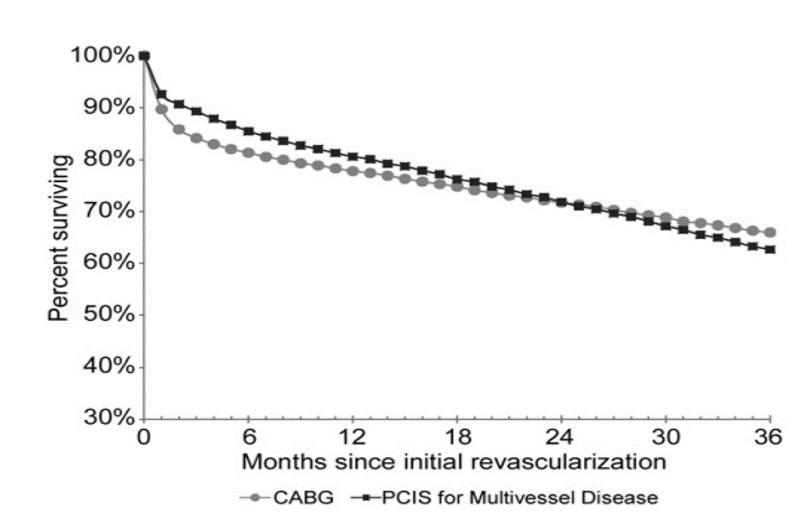


**Supplementary Figure 6.** Test of proportional hazard assumption (A) and in log–log survival plots (B).

**A**

**
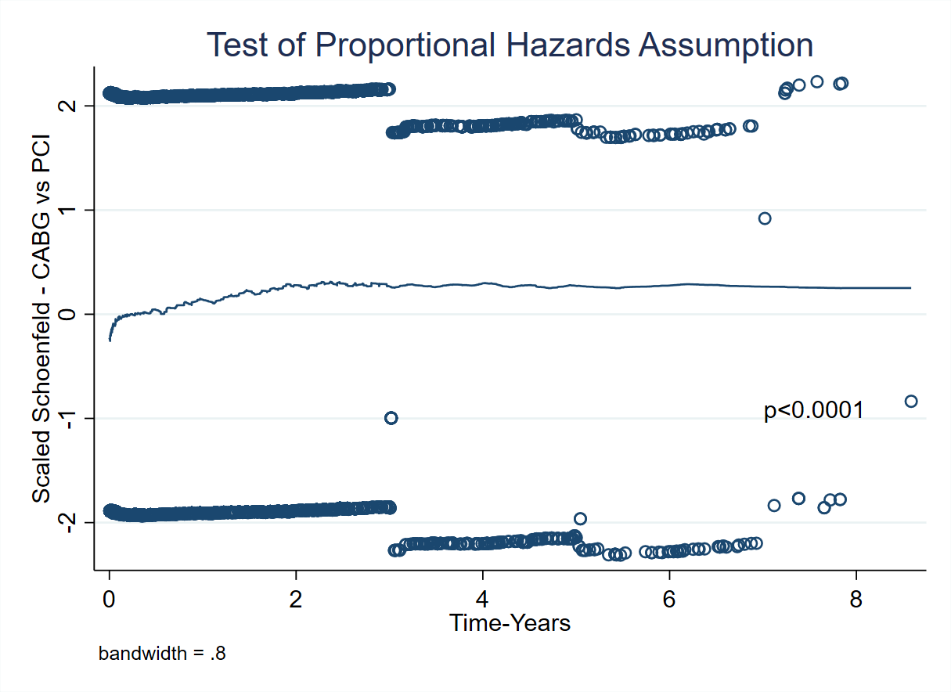
**

**B**

**
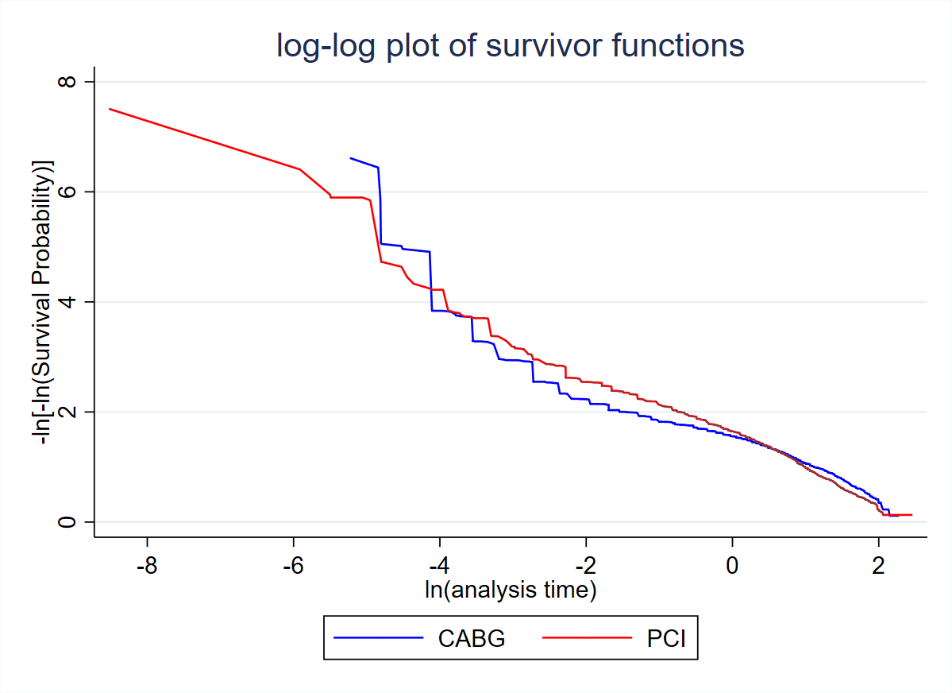
**

**Supplementary Figure 7.** Forest plot for perioperative all-cause mortality.

**
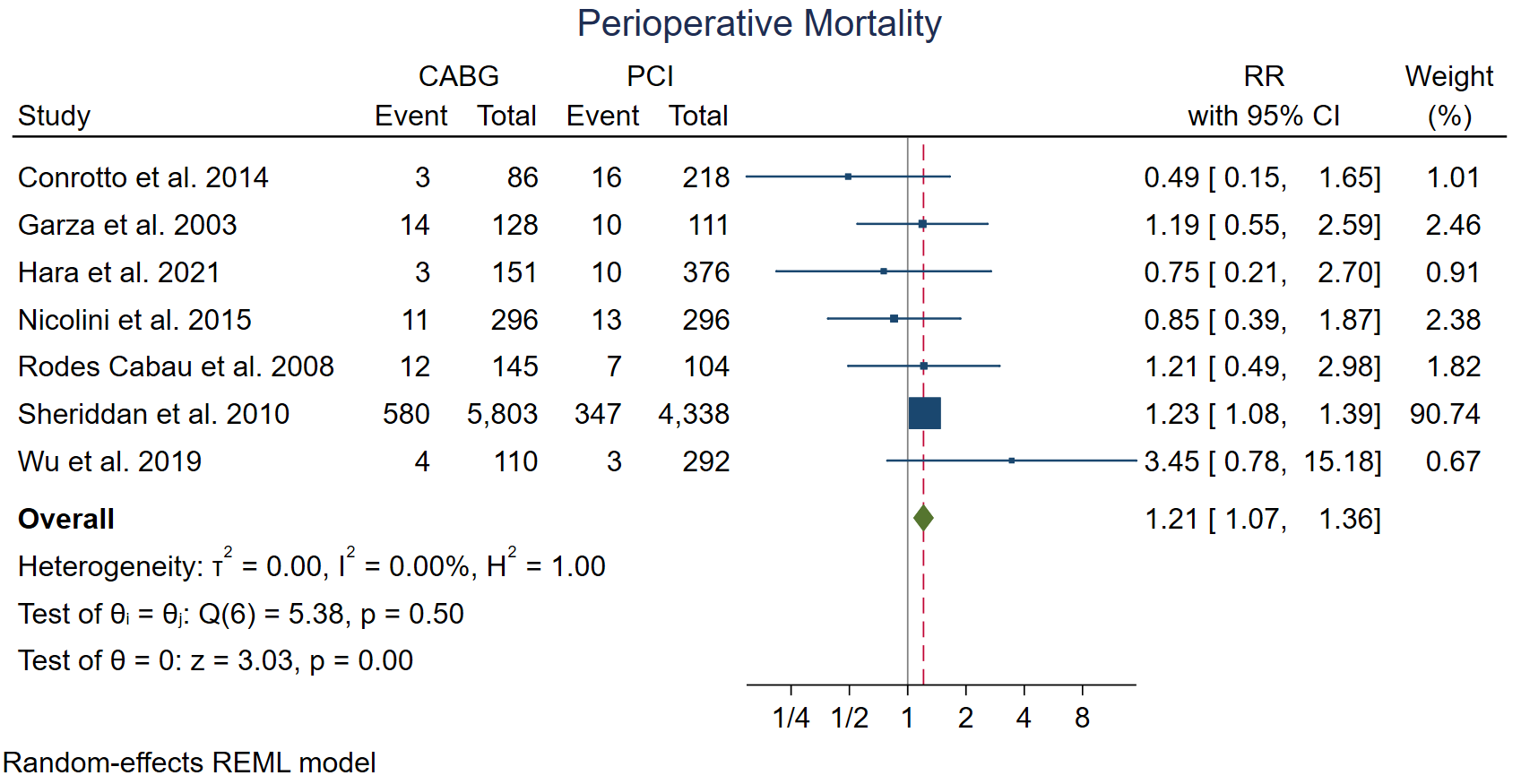
**

**Supplementary Figure 8.** Forest plot for acute renal failure.

**
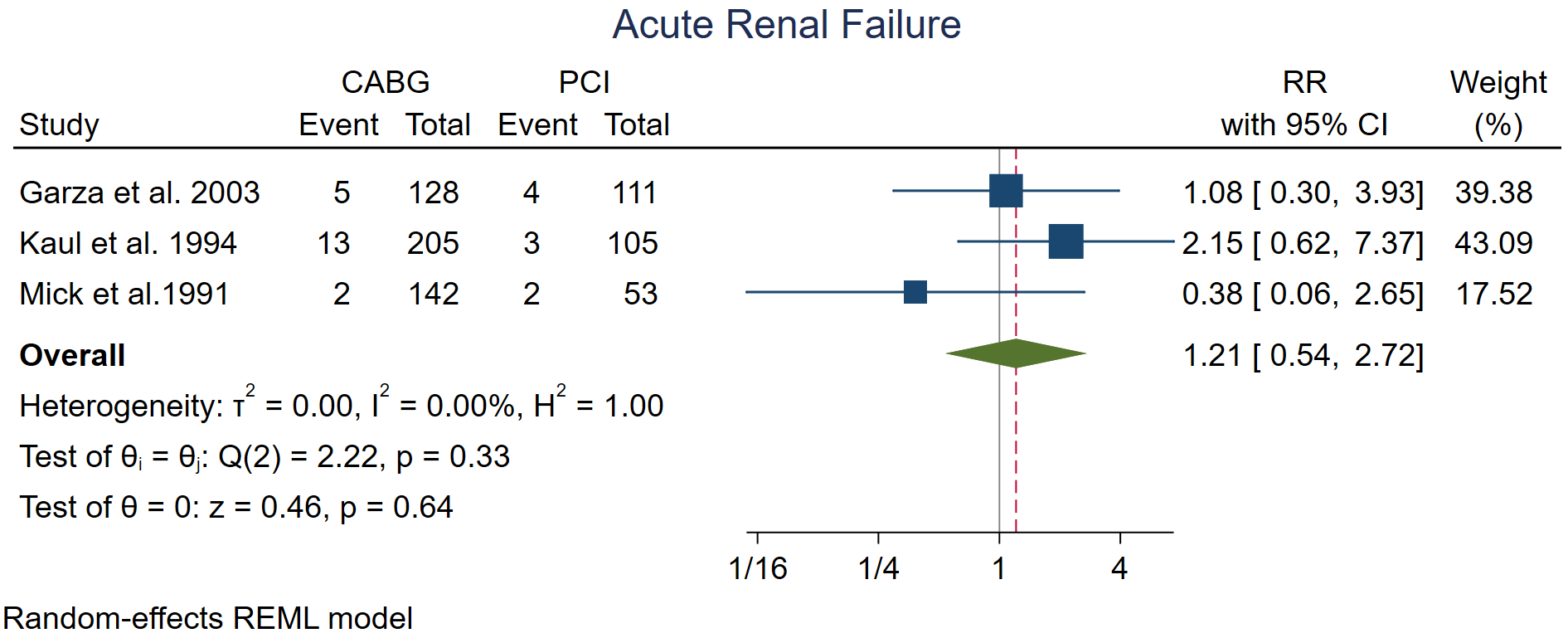
**

**Supplementary Figure 9.** Forest plot for re-revascularization.

**
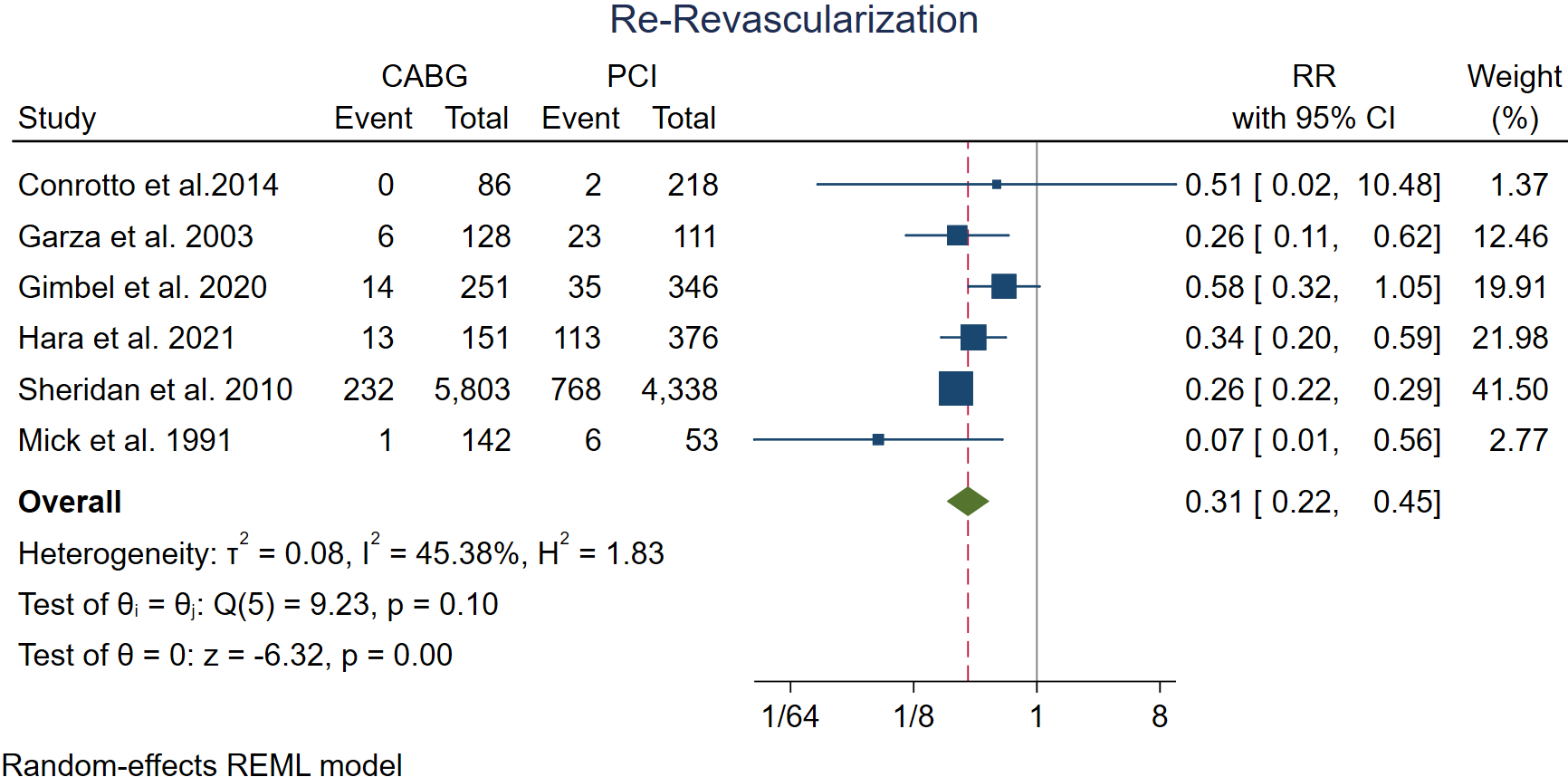
**

**Supplementary Figure 10.** Forest plot for stroke.

**
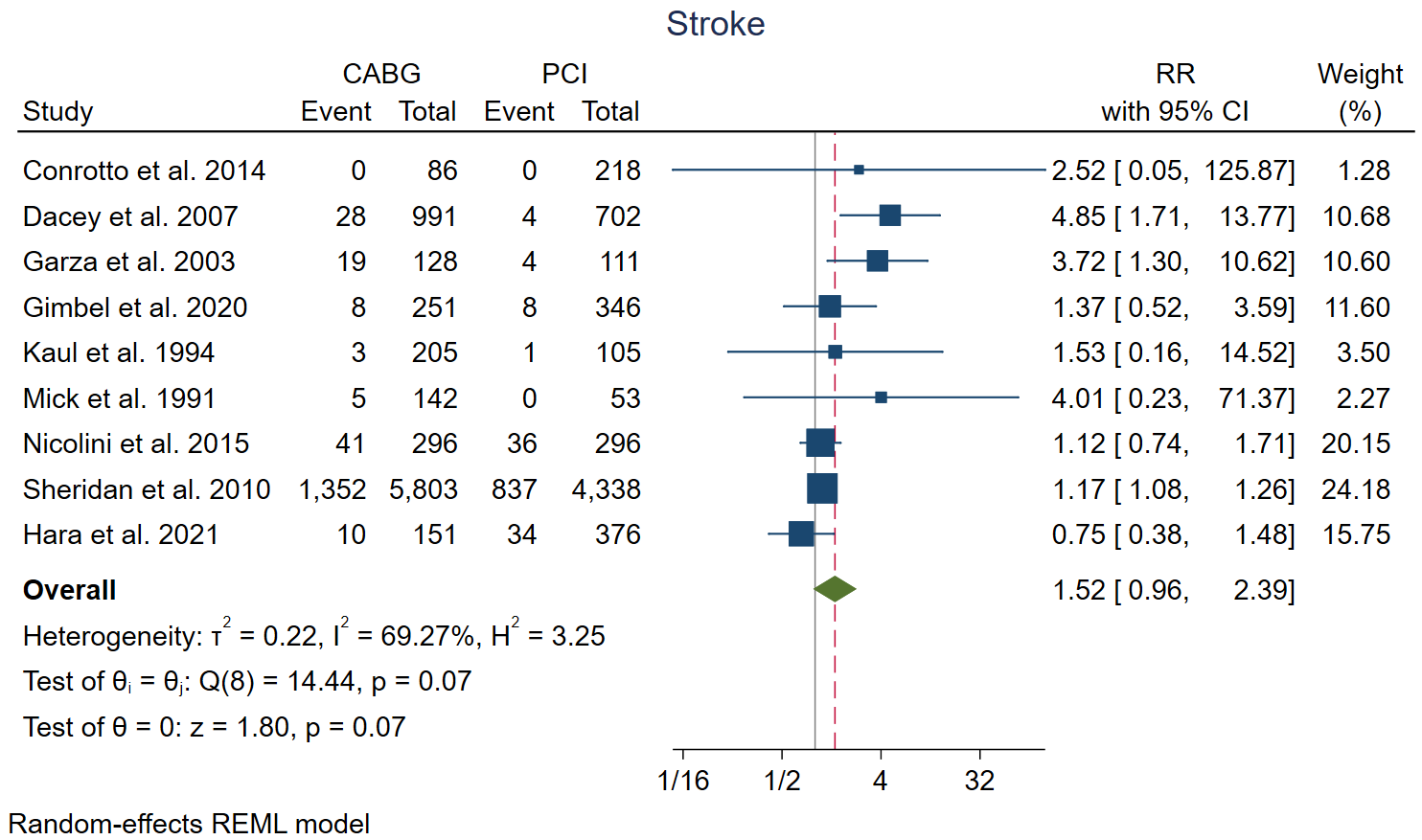
**
